# Molecular prospecting for cryptic species of the *Hypholoma fasciculare* complex: toward the effective and practical delimitation of cryptic macrofungal species

**DOI:** 10.1038/s41598-020-70166-z

**Published:** 2020-08-06

**Authors:** Hirotoshi Sato, Ryoma Ohta, Noriaki Murakami

**Affiliations:** 1 Graduate School of Human and Environmental Studies, Kyoto University, Sakyo, Kyoto, 606-8501 Japan; 2 Makino Herbarium, Tokyo Metropolitan University, Hachioji, Tokyo 192‐0397 Japan

**Keywords:** Fungal biology, Fungal ecology, Fungal evolution, Population genetics, Taxonomy

## Abstract

Many macrofungal cryptic species remain unidentified. A possible solution is to increase the number of loci analyzed and use rigorous statistics for macrofungal species delimitation. To validate this assumption, cryptic species of the *Hypholoma fasciculare* complex, a group of common wood-decomposing fungi, were attempted to be delineated. Massively parallel sequencing of mitochondrial ribosomal RNA (mt_rRNA), nuclear ribosomal internal transcribed spacer (ITS) region, and 24 single-copy genes were performed for 96 specimens collected in Japan. Then, the species boundaries were inferred using comparative gene genealogies (mt_rRNA vs. ITS), Bayesian Poisson tree process (bPTP) model for the phylogeny of concatenated nuclear sequences, and analysis of molecular variance (AMOVA) for single nucleotide polymorphisms. In both the mt_rRNA and ITS phylogenies, the *H. fasciculare* complex was not divided into well-supported clades. Nevertheless, based on the bPTP, two mitochondrial haplotypes were inferred to represent distinct species (*H. fasciculare* and *H. subviride*). The results of AMOVA also indicated that the differentiation of nuclear loci can be explained mostly by differences between haplotype. These results suggest that it is necessary to increase the number of target loci to 20 or more and use both phylogeny-based and population genetics-based statistics for the accurate delimitation of macrofungal species.

## Introduction

Accurate species delimitation is fundamental for understanding species diversity, evolutionary diversification, ecology and biogeography of fungi. So far, about 140,000 fungal species have been reported worldwide (https://www.catalogueoflife.org/), but the vast majority of fungal taxa are still considered to be unknown. Indeed, the total number of fungal species worldwide is estimated to be 1.5 million^1^, 3.5 to 5.1 million^2^, or 2.2 to 3.8 million^3^. The fact that relatively few fungal species have been documented can be primarily attributed to the limited number of taxonomically relevant morphological characteristics of fungi; this is true even of macrofungi, which form conspecific fruit bodies^4–7^. Therefore, better methods for the identification of cryptic fungal species (i.e., reproductively isolated populations of fungi that converge phenotypically and are not visibly different) are required for improved fungal species recognition.

Use of DNA sequencing, specifically multilocus sequencing, has made it possible to distinguish cryptic species of any fungal taxa without mating tests, which cannot be applied to unculturable fungi. The nuclear ribosomal DNA internal transcribed spacer (ITS) region has been used as a universal barcode for fungi because of the availability of universal primers^8,9^ and because it indicates inter- and intraspecific variation most clearly^10^. Fungal species can be roughly delimited based on their molecular phylogeny, inferred from ITS sequences, and molecular operational taxonomic units (MOTUs), identified based on similarities among ITS sequences^10,11^. However, a single molecular marker is not necessarily sufficient for accurate species delimitation^12,13^. Therefore, approaches that incorporate many loci are necessary for effective detection of species boundaries.

However, methods for accurate species delimitation of fungi based on multiple loci have not been well developed. Over the last three decades, comparative gene genealogies (i.e., visual inspections of individual gene genealogies^14^) have been used to delimitate fungal species, including pathogenic fungi^6,15–18^, lichenized fungi^19–21^, and macrofungi^7,22,23^. This method clearly differs from single-locus phylogeny because distinct linkage disequilibrium among different loci indicates the restriction of gene flow, which is an important indication of reproductive isolation among sympatrically distributed fungal taxa^7,17^. Nevertheless, this method has several limitations. First, there is a lack of rigorous statistical frameworks for detecting species boundaries; bootstrap supports are an exception, although they represent the reliability of nodes rather than that of species boundaries. This becomes especially problematic when trying to delimit recently diverged species, for which reciprocal monophyly is not necessarily demonstrated across multiple loci^24,25^. Second, only a small number of loci can realistically be used in this method because of the substantial amount of labor required to use many loci. Third, heterozygotes are not taken into account in this procedure despite the fact that they could provide fundamental information for inferring the amount of gene flow in populations^26^. Thus, the methodologies for detecting macrofungal species using multiple loci could be considerably improved.

Notably, several studies have developed statistical frameworks for exploring and testing species boundaries based on molecular phylogenetic trees. For instance, the General Mixed Yule Coalescent (GMYC) model^27^ and its Bayesian implementation^28^ (bGMYC) separate the branching patterns observed in a time-calibrated ultrametric tree into two events: speciation events between species-level taxa (modeled by a Yule process) and coalescent events between lineages sampled from within species (modeled by the coalescent). The GMYC model assumes that the coalescence process is far more common than the speciation processes within the tree and attempts to determine a threshold that reflects the transitions between both processes. The Poisson tree processes (PTP) model and its Bayesian implementation (bPTP) are similar to the GYMC model^29^, and are often integrated with the evolutionary placement algorithm (EPA), in which short reads are placed into a given reference tree obtained from full-length sequences to determine the evolutionary origin of reads^30^. The PTP model assumes that the number of substitutions between species is significantly higher than the number of substitutions within species, although they differ from the GMYC model in that they use the number of substitutions to directly model the speciation rate. The application of these methods to concatenated multilocus sequences could provide rigorous statistical frameworks for distinguishing species, although heterozygotes would still not be distinguished from homozygotes.

Meanwhile, analysis of population genetics using single nucleotide polymorphisms (SNPs) is a powerful method of detecting reproductively isolated species. This method has some advantages over phylogeny-based species delimitation by testing distinct linkage disequilibrium among different loci. Specifically, the use of SNP markers allows heterozygotes to be distinguished from homozygotes. Additionally, statistical frameworks, such as Wright's *F* statistics and its analogs, can be implemented to utilize allele frequency data to quantify population subdivision and estimate the amount of gene flow^26^. An approach based on population genetics can therefore provide reliable evidence for reproductive isolation among fungal lineages, although caution should be exercised when delimiting allopatric species^24,31^. Although less common than phylogeny-based species delimitation, population genetics analysis has been used to delimit the species boundaries of various fungi^32–35^.

**Figure 1:**
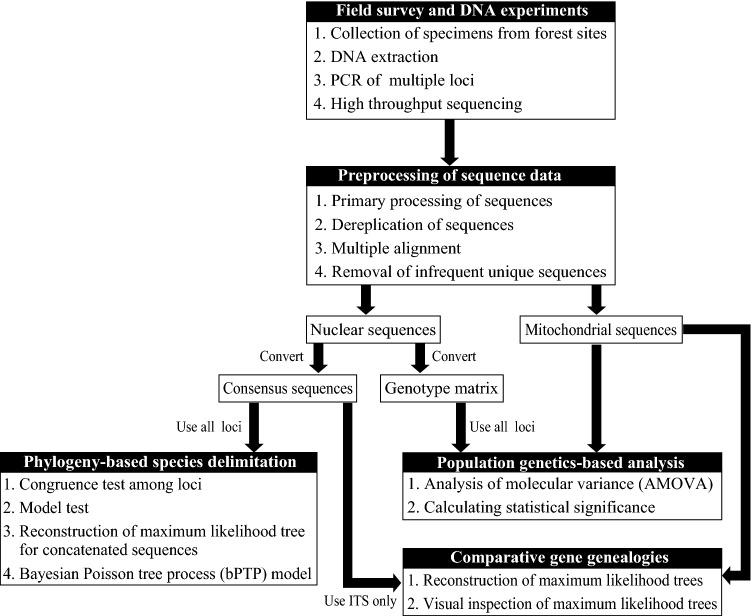
A brief outline of three DNA-based analyses that are used for inferring species boundaries of the *Hypholoma fasciculare* complex in this study.

A fundamental question addressed in the present study is how increasing the number of loci analyzed (i.e., using 20 or more loci) and using two different statistical frameworks (i.e., both phylogeny-based and population genetics-based species delimitation) could improve the accuracy of the delimitation of closely related cryptic macrofungal species. To explore this question, species boundaries were examined in the species complex of *Hypholoma fasciculare* (Strophariaceae, Agaricales). *Hypholoma fasciculare* is a common and widespread wood-decomposing fungal species, which is distinguished by a sulfur-yellow pileus with an orange-brown center, crowded gills that are initially yellow but darken as the blackish-purple spores drop, and the very bitter taste of their basidiomes^36–39^. To date, the taxonomy of this species remains to be resolved. Because variations of basidiome sizes and colors were reported in this species, the fungal specimens identified as *H. fasciculare* could cluster into several distinct species^37^. Moreover, *H. fasciculare* could be confused with *H. subviride*, which has been reported only around the Central America but is morphologically similar to *H. fasciculare*^40,41^. To identify cryptic species in the *H. fasciculare* complex in Japan, the large (mtLSU) and small (mtSSU) subunits of mitochondrial ribosomal RNA, the nuclear ITS1 and ITS2 region, and 24 nuclear single-copy genes were sequenced for the *H. fasciculare* complex using the massively parallel sequencing technique. Thereafter, species within the complex were tried to be delimited using (1) comparative gene genealogies of the concatenated mtLSU and small mtSSU regions (mt_rRNA dataset) and the concatenated ITS1 and ITS2 regions (ITS dataset), (2) the bPTP model that was applied to the molecular phylogeny inferred from the concatenated nuclear sequences (nuc_concat dataset), and (3) hierarchical analysis of molecular variance (AMOVA) based on nuclear SNPs. An outline of these analyses is shown in Fig. 1. The empirical study of the *H. fasciculare* complex should show the importance of increasing number of loci analyzed and using rigorous statistical frameworks for the accurate detection of cryptic macrofungal species.

## Results

### Molecular phylogenetic inference

**Figure 2:**
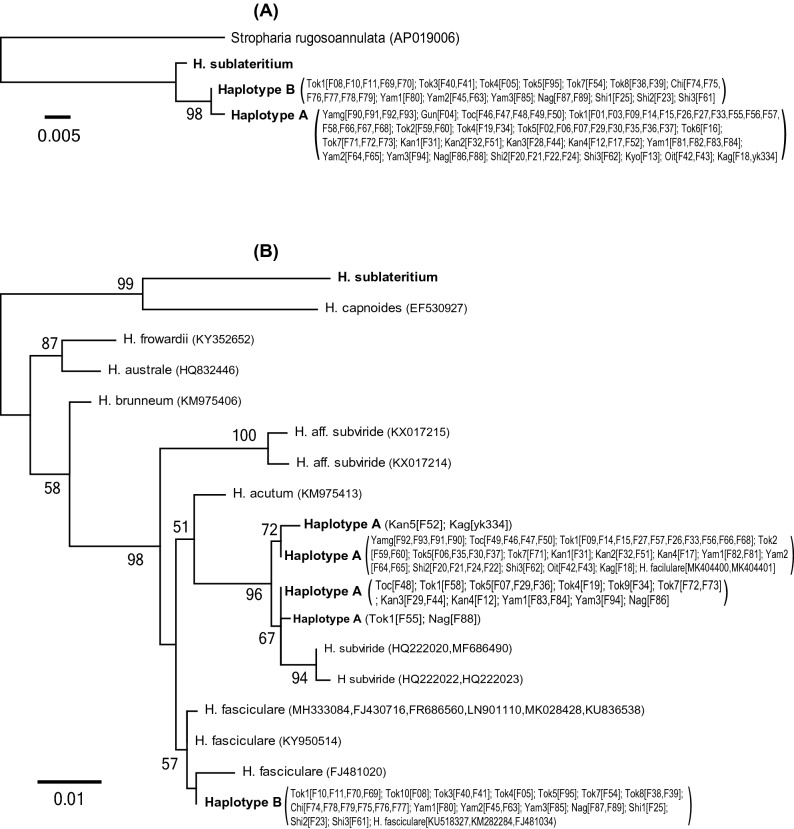
Maximum-likelihood (ML) trees of the *Hypholoma fasciculare* complex, as inferred from the nucleotide sequences of (**A**) mitochondrial ribosomal RNA (mt_rRNA) and (**B**) nuclear ribosomal internal transcribed spacer (ITS) region. The numbers near the branches are bootstrap values (> 50%). Samples with identical sequences are pre-merged as unique sequences. Each taxon name indicates locality ID and strain name (i.e., Locality ID [Strain names]), as listed in Table S1. Bold names indicate that sequences are determined in this study.

The mt_rRNA dataset consisted of 700 nucleotide sites (mtLSU, 301 bp; mtSSU, 399 bp), of which 61 were variable. Samples of the *H. fasciculare* complex in Japan were divided into two mitochondrial haplotypes (hereafter, haplotype A and B; Fig. 2A). The nuclear ITS dataset consisted of 676 nucleotide sites (ITS1, 310 bp; ITS2, 366 bp), of which 87 were variable. In the ML tree of the ITS dataset (Fig. 2B), samples of haplotype A were divided into four unique sequences, one of which was identical to sequences of *H. fasciculare* obtained from the International Nucleotide Sequence Database (INSD) (MK404400 [China] and MK404401 [China]). These four unique sequences and two unique sequences of the INSD representing *H. subviride* (HQ222020 [USA], MF686490 [USA], HQ222022 [Costa Rica], and HQ222023 [Belize]) formed a clade that was supported by a 96% bootstrap value (Fig. 2B). Pairwise phylogenetic distances between unique sequences of this clade were relatively small (Table 1). In the ML tree of the ITS dataset, all samples that were assigned to haplotype B were merged into a single unique sequence (Fig. 2B) that was identical to the INSD sequences identified as *H. fasciculare* (KU518327 [China], KM282284 [India], and FJ481034 [China]). Pairwise phylogenetic distances from the unique haplotype B sequence to three unique sequences in the INSD identified as *H. fasciculare* (MH333084 [Mexico], FJ430716 [Czech Republic], FR686560 [Germany], LN901110 [Czech Republic], MK028428 [Switzerland], KU836538, KY950514 [China], and FJ481020 [China]) were somewhat smaller than those to the haplotype A and *H. acutum* (Table 1). The phylogenetic relationships among these groups remained unclear (Fig. 2B).

**Table 1:** Phylogenetic distance between *Hypholoma* taxa calculated based on the maximum likelihood tree, as inferred from the nucleotide sequences of nuclear ribosomal internal transcribed spacer (ITS) region.

	Haplotype A	Haplotype B	*H. fasciculare*	*H. subviride*	*H. acutum*
Haplotype A	0.0015–0.0075	0.0187–0.0219	0.0181–0.0286	0.0046–0.0142	0.0189–0.0222
Haplotype B		0	0.0016–0.0060	0.0225–0.0278	0.0109
*H. fasciculare*			0.0016–0.0093	0.0200–0.0339	0.0098–0.0157
*H. subviride*				0.0017	0.0223–0.0296
*H. acutum*					0

Taxon names correspond to those shown in Fig. 2B

**Figure 3:**
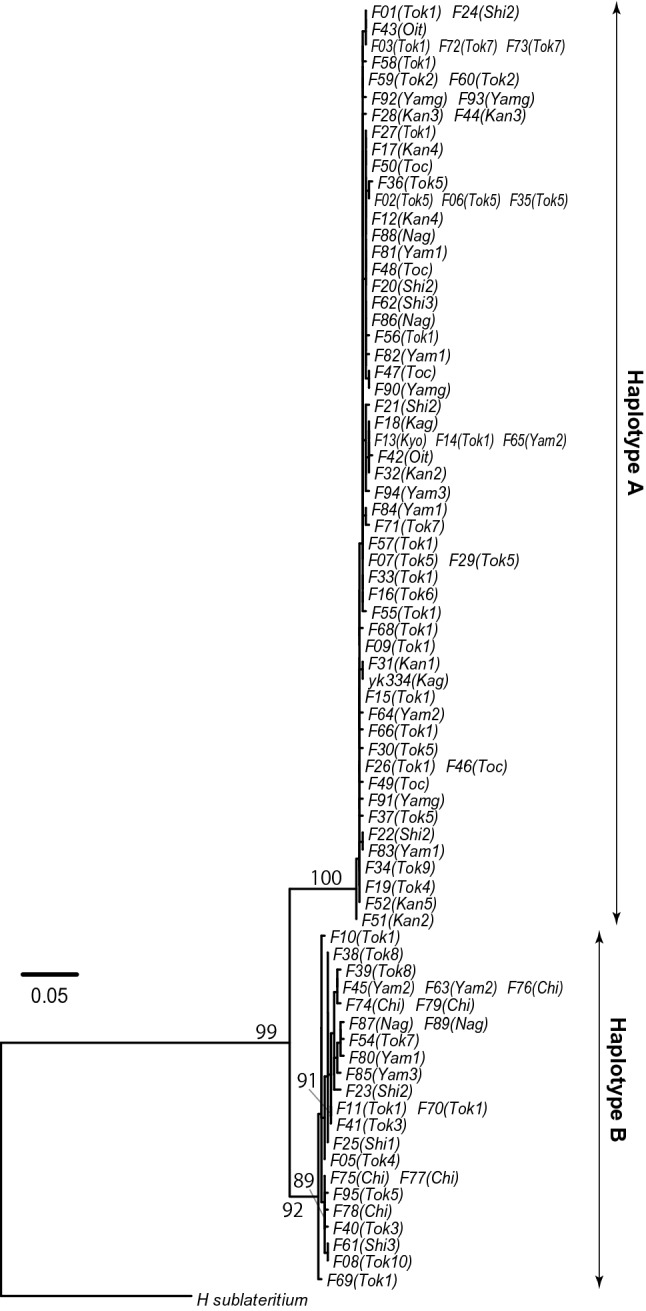
Maximum-likelihood (ML) trees of the *Hypholoma fasciculare* complex, as inferred from the nucleotide sequences of concatenated nuclear genes (nuc_concat). Nuclear genes comprise nuclear ribosomal internal transcribed spacer (ITS) region and 20 single-copy genes. Haplotypes A and B correspond to two unique sequences of mitochondrial ribosomal RNA (mt_rRNA) region identified in the *H. fasciculare* complex. The numbers near the branches are bootstrap values (> 50%). Samples with identical sequences are pre-merged as unique sequences. Each taxon name indicates locality ID and strain name (i.e., Strain names (Locality ID)), as listed in Table S1.

The nuc_concat dataset consisted of 6,680 nucleotide sites, of which 1,463 were variable. In the ML tree of the nuc_concat dataset, samples of haplotype A and B formed distinct clades that were supported by 100% and 92% bootstrap values, respectively (Fig. 3). Pairwise phylogenetic distances within the clades of haplotype A and B ranged from 0.0000 to 0.0056 and from 0.0002 to 0.0090, respectively. Pairwise phylogenetic distances between the clades of haplotype A and B ranged from 0.0337 to 0.0554.

### EPA and bPTP

**Figure 4:**
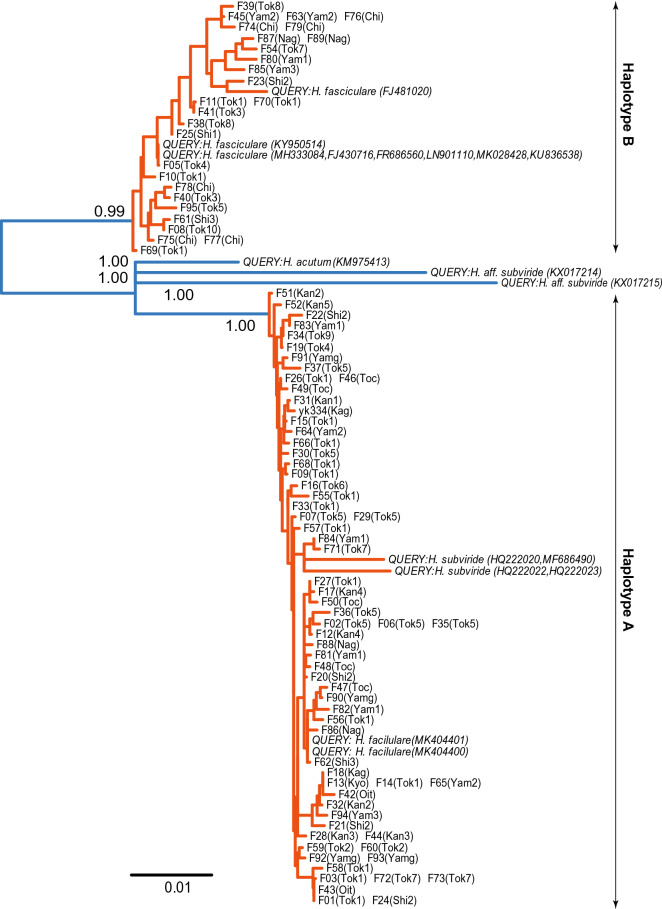
Results for the integrated analysis of evolutionary placement algorithm (EPA) and the Bayesian Poisson tree process (bPTP) model for the *Hypholoma fasciculare* complex. Samples with identical sequences are pre-merged as unique sequences. Each taxon name indicates locality ID and strain name [i.e., Strain names (Locality ID)], as listed in Table S1. Italicized samples correspond to the queries of EPA, for which sequences of nuclear ribosomal internal transcribed spacer (ITS) are obtained from the international nucleotide sequence database (INSD). Orange and blue lines indicate that differences are inferred as intraspecific and interspecific differences by bPTP model, respectively. The numbers near the branches are posterior probabilities (> 0.5) that the nodes represent species delimitation.

Based on the EPA, all ITS queries for *H. fasciculare* were placed within the clade of haplotype B, except for two queries (MK404400 [China] and MK404401 [China]) (Fig. 4). The ITS queries for *H. subviride* were placed within the clade of haplotype A (Fig. 4). The ITS queries for *H. acutum* and *H.* aff. *subviride* were placed outside the clades of both haplotype A and B (Fig. 4).

According to the bPTP model, the posterior probabilities that the clades of haplotype A and B represent distinct species were 0.99 and 1.00, respectively. In addition, three additional groups were inferred as species by the bPTP with 1.00 posterior probabilities. These groups contained the unique sequence of *H. acutum* and two unique sequences of *H.* aff. *subviride*.

### Population genetics analysis

AMOVA revealed significant genetic differentiation in nuclear loci between two mitochondrial haplotypes, explaining 78.8% of the total variance of nuclear loci (Φ = 0.788, *P* < 0.001; Table 2). The remaining variation of nuclear loci was explained by differentiation among geographical regions within the mitochondrial haplotypes (2.1%, Φ = 0.100, *P* < 0.001), between samples (1.3%, Φ = 0.068, *P* = 0.092), and within samples (i.e., heterozygotes; 17.8%, *P* < 0.001) (Table 2). These results were almost the same if the cut-off levels of samples and loci were changed.

**Table 2:** Results for analysis of molecular variance (AMOVA) based on single nucleotide polymorphism of nuclear loci, considering haplotypes, regions (sampling locality) and individuals (samples).

Variation source	Df	Sum of squares	Mean squares	% of variance	*Φ*	*P*
Between Haplotypes	1	17,831.1	17,831.1	78.8	0.788	0.000
Between Regions Within Haplotype	36	3,199.8	88.9	2.1	0.100	0.000
Between Samples Within Region	54	3,242.1	60.0	1.3	0.068	0.092
Within Samples	92	4,823.1	52.4	17.8	–	0.000
Total	183	29,096.0	159.0	100.0	–	–

AMOVA is a hierarchical analysis of molecular variance, estimating the % of molecular variance accounted for by each level of the nested sampling hierarchy as well as *Φ* (≒*F*st).

### Observation of morphologies and habitats

**Figure. 5:**
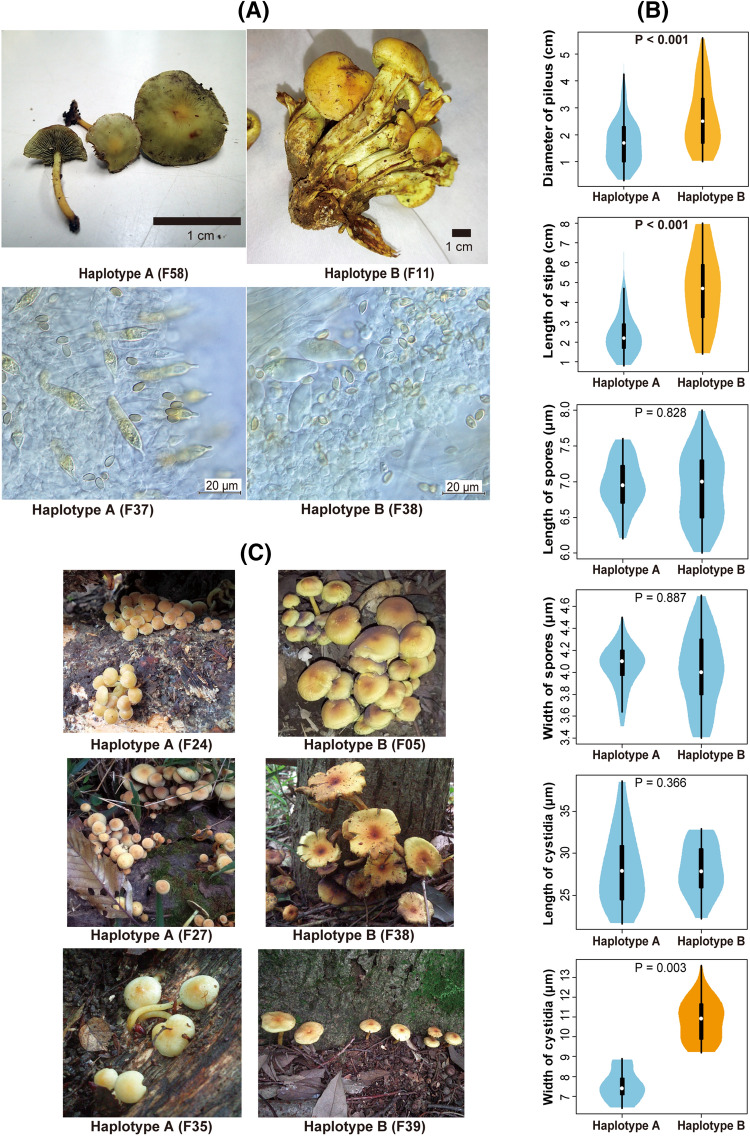
Macroscopic and microscopic features of the *Hypholoma fasciculare* complex. (**A**) Photographs of basidiomes, basidiospores and gill cystidia, below which haplotype and strain ID of each sample are shown. (**B**) Difference of sizes in pileus, sitipe, basidiospores and gill cystidia between haplotypes A and B. The box plot displays the median ("white circle"), first and third quartile ("black rectangle"), and full range of variation (from min to max; "solid line"). The violin plot displays the distribution of features. Different colors in violin/box plots indicate significant differences in Mann–Whitney U test. *P* values are shown above violin/box plots. (**C**) Growth environments of haplotypes A and B.

Differences in macroscopic and microscopic characteristics were observed between haplotypes A and B. The diameter of the pileus was significantly greater in haplotype B (1.0–5.6 cm, mean = 2.7 cm) than in haplotype A (0.3–4.9 cm, mean = 1.8 cm), although variation within haplotypes was fairly large (Fig. 5A,B). Similarly, the length of the stipe was significantly greater in haplotype B (1.4–8.0 cm, mean = 4.7 cm) than in haplotype A (0.7–0.6.6 cm, mean = 2.4 cm) (Fig. 5A,B). The length and width of the basidiospores was almost the same between haplotypes A (6.2–7.6 μm [mean = 7.0 μm] × 3.5–4.5 μm [mean = 4.0 μm]) and B (6.0–8.0 μm [mean = 6.9 μm] × 3.4–4.7 μm [mean = 4.0 μm]) (Fig. 5A,B). Although the length of the cystidia on the gill (cheilo- and pleurocystidia) was almost the same in haplotype A (21.6–38.6 μm; mean = 28.2 μm) as in haplotype B (22.2–32.9 μm; mean = 28.1 μm), its width was significantly greater in haplotype B (9.2–13.6 μm; mean = 10.8 μm) than in haplotype A (6.4–8.9 μm; mean = 7.5 μm) (Fig. 5A,B).

In the present study, only a part of the qualitative data on the habitat of *H. fasciculare* complex was available. However, it appears that fruit bodies of haplotype A occurred directly on stumps or fallen trees, whereas those of haplotype B occurred on soil or around the bases of dead trees (Fig. 5C).

## Discussion

Our results provide important information for taxonomy of the *Hypholoma fasciculare* complex. Moreover, the present findings suggest that the use of rigorous statistical methods, such as phylogeny-based and population genetics-based analyses, for multilocus datasets allows for effective and practical detection of cryptic fungal species that cannot be easily distinguished by single gene genealogy and comparative gene genealogies.

The species boundaries in the *H. fasciculare* complex remained unclear when analyzed solely using the comparative gene genealogies of mt_rRNA and/or ITS sequences, presumably due to the insufficient taxonomical and phylogenetic resolution of single-locus phylogeny (Fig. 2). In general, distinct species do not necessarily form distinct clades with high bootstrap supports in mitochondrial phylogenies because of the insufficient variations in mitochondrial genes^10,42,43^. This problem appears to lie in the mt_rRNA phylogeny of the *H. fasciculare* complex. Additionally, intra- and interspecific variation could not be easily distinguished based on the phylogeny of the ITS region. These results highlight the limitations of single-locus phylogeny for distinguishing species, as suggested for other organisms^24,25^.

In contrast, use of bPTP model and AMOVA with 20 or more nuclear loci resulted in reasonably successful species delimitation of the *H. fasciculare* complex of Japan. The results of the bPTP model suggest that differences in mitochondrial sequence correspond to the species boundaries of the *H. fasciculare* complex (Fig. 4). Additionally, AMOVA for nuclear SNPs suggests that fungi with different mitochondrial haplotypes are reproductively isolated (Table 3). Specifically, distinct cytonuclear disequilibrium (i.e., linkage between cytoplasmic and nuclear markers^44,45^) appeared to occur even in regions where both haplotypes coexist (e.g., Tok1, Yam1, and Yam2). Furthermore, populations consisting of fungi with the same haplotypes did not appear to deviate from the Hardy–Weinberg equilibrium (Table 3). Overall, both bPTP and AMOVA support the theory that the *H. fasciculare* complex in Japan should be divided into two species.

**Table 3:** Forward (F) and reverse (R) PCR primers to amplify a short fragment of each region.

Gene	Forward primer (5′–3′)	Reverse primer (5′–3′)
Pol30 (FG546)^b^	CARGCNATGGAYAACTCYCAYGT	TCRATRTCCATNAGYTTCAT
Gdi1 (FG576)^b^	AAGAAGGTSCTYCACATGGA	GCYTCCATYTCBGTRCTBGG
Hem2 (FG652)^b^	AARTCSATGYTCATGTAYCC	GTGTAYTCGCAVAGRCAVACRTC
Ade12 (FG673)^b^	AGCATCGGNACMACVAAGAA	CCRAARTCRATRTCVAGCAT
Pup1 (FG684)^b^	GCVGACAAGAACTGYGARAAGG	CCGTGWGGRTGGATNGTRAA
Hem15 (FG756)^b^	CAGTAYCCBCARTAYAGYTGYAG	TCYCCRCGGTTBACVACYGACAT
Rfc4 (FG761)^b^	AAYGTVTTCAAAGTBTGYGAYCA	CATRTGYGTRAABCCRATYTCCT
Arc40 (FG771)^b^	TGATCACNTCNATYGAYTGGGC	GTCGAYCKGATNGGYTTCTT
Prp46 (FG813)^b^	AARGTYATHCGGCAYTAYCAYGG	CGVACAGAYTTYTTGTGRTG
Uba1 (FG848)^b^	GARTTYGAGAAGGAYGAYGA	GGYTCNGAGAARCCRAAGAA
Rrb1 (FG927)^b^	GARGGBTTYGCNATGGAYTGGGC	TTCCARCTRATVACATTNACRTC
Sac6 (FG975)^b^	GAGCTBGAVGAYTGGGTHGAGGT	KRCABTCGTCRAAGAKYTGCAT
Gsh1 (MS320)^b^	AAYCCWCATGCNCGHTTYCCGT	CARCADCCCATBCCRAARCCCAT
Coq5 (MS348)^b^	GTSCACMGGYTVTGGAARGA	CCRAAWGCRATGGTRTAVAGGTC
Trp2 (MS353)^b^	TACATGTTYTAYYTBGAYTGYGG	GTYARRTGRATGACRTGRCTGAA
Lat1 (MS355)^b^	GTYGAYATCAACATGGRYAAG	CCRTCGCGVGCCTTCTTBGCRAG
Ala1 (MS358)^b^	TTCGARATGYTSGGMAAYTGGTC	GGKCCRGTYGCDCCCATYTC
Brx1 (MS361)^b^	TGGGCTGCRAARACRCCNAAYGG	TGGAARTTNCGGAACCADATYTT
Kog1 (MS378)^b^	TGTGYATYGCGCARATVTGGG	TCYTCCCARTAGAKCCANGCRCA
Ppt1 (MS417)^b^	AAYCAYGARGCRAARGABATGAA	GTRACRCCRTCYTTRCTGAA
Ils1 (MS444)^b^	GAYGGMAAGAARATGAGCAARAG	AGRATCCAKCGRTCCATVACRTT
Ded81 (MS453)^b^	GGCAGATGACNGAYATCATYGG	CGCTGRTCRGTRWACCARTARTA
Mvd1 (FG1021)^b^	TCVYRCAACAACTTCCCYAC	GTCTCGACGGTGCGYTGCATVCC
Pwp2 (FG1024)^b^	CAYTWYTTCGAYATGAACAC	AGCTTYCCHGTYTGYACVGACCA
ITS1^c^	TAGAGGAAGTAAAAGTCGTAA	TTYRCTRCGTTCTTCATC
ITS2^c^	GATGAAGAACGYAGYRAA	RBTTTCTTTTCCTCCGCT
mtLSU^a^	CCGTACCCTAAACCGACACA	TTAAGACCGCTATTAACCA
mtSSU^a^	CAATGCTCGCAAGAGTGAAC	TTCGTTYCTCAGCGTCRATC

These primers contain an overlapping region of the Illumina sequencing primers and 6-mer Ns.

^a^Primers designed in this study; ^b^predesigned primers^15^; ^c^predesigned primer^8^.

It was also shown in this study that two species in the *H. fasciculare* complex could be distinguished morphologically and ecologically (Figs. 5). The species represented by haplotype B is characterized by its relatively large pileus, long stipe, and wide gill cystidia compared to the species represented by haplotype A. It should be highlighted that these diagnostic features would not have been easily distinguishable from intraspecific morphological variation without the aid of DNA-based approaches. In other words, DNA-based species delimitation is not a substitute for the detection of diagnostic morphologies, but rather an efficient tool for detecting diagnostic morphologies. Interestingly, the habitats where the fruit bodies of the fungi studied occur appear to differ between the two species, implying that the species differ in the substrates that they decompose. This finding goes against the assumption that closely related fungal species have similar physiological features. Although further surveys, for example the analysis of stable isotopes^46,47^, are required to verify the divergence of trophic status, our results suggest morphological and ecological differentiation between two species in the *H. fasciculare* complex.

Our results indicate the necessity for revising the nomenclature of the *H. fasciculare* complex. Although the type specimens of *H. fasciculare* were lost, several evidences indicate that haplotype B corresponds to *H. fasciculare*. First, the diameter of the pileus described originally is somewhat similar to that of haplotype B^48^. Second, the type locality of *H. fasciculare* (supposedly the United Kingdom, home of the nomenclator, or its surrounding countries) seems to be included in the geographical distribution of haplotype B rather than in that of haplotype A, because all of the fungal materials of *H. fasciculare* complex collected in European countries were inferred to be conspecific to the samples of haplotype B based on integrated analysis using EPA and bPTP (EPA-bPTP) (Fig. 4). Meanwhile, haplotype A seems to correspond to *H. subviride*, which is morphologically similar to *H. fasciculare*^41^. Our results indicate that haplotype A is conspecific with the fungal materials identified as *H. subviride* in the INSD, which were collected in North and Central America, near to the type locality of *H. subviride* (i.e., Cuba) (Fig. 4). Further, macroscopic morphologies of haplotype A do not contradict the original description of *H. subviride*^49^. In summary, haplotypes A and B should be treated as *H. subviride* and *H. fasciculare*, respectively.

Notably, our results indicate that increasing number of loci analyzed is generally useful for accurate species delimitation of macrofungi. The molecular phylogeny based on 22 concatenated nuclear loci (i.e., the nuc_concat dataset) appeared to increase the variable sites and thereby improve taxonomic and phylogenetic resolution. Genome-wide analyses, such as restriction-site associated DNA sequencing^50^ (RAD-seq) and multiplexed inter-simple sequence repeat genotyping by sequencing^51^ (MIG-seq) can provide many more loci and thus are more useful tools for population genetic/genomic studies. However, these two methods are limited in the utility at deeper phylogenetic scales: few orthologous loci are typically recovered across disparate taxa in the former method^52^ and the level of homoplasy is expected to increase with increasing the time of divergence between populations in the latter method^53^. Thus, they are not necessarily suitable for detecting cryptic macrofungal species as phylogenetically distinct fungal species in addition to recently diverged species are often confused owing to their morphological similarities^7,23^. Therefore, sequencing of 20 or more loci, as carried out in the present study, seems to be efficient and effective for the detection of cryptic fungal species.

The use of adequate statistical methods for the phylogeny-based approach is fundamental for improving the accuracy of macrofungal species delimitation. Use of bPTP allows the speciation process to be distinguished from the coalescent process based on the branch length of molecular phylogeny, thereby yielding more reliable detection of species boundaries than a visual inspection of molecular phylogeny^29^. Use of the EPA is also useful for understanding the phylogenetic placement of fungal materials for which only short reads are available in the INSD^30^. The bPTP model is almost equivalent to the GYMC model^27^ and its Bayesian implementation^28^ (bGMYC), which require a time-calibrated ultrametric tree. However, it is important to note that substantial computation time is required to reconstruct an ultrametric tree and the use of GMYC together with EPA is somewhat difficult. Therefore, the use of EPA-bPTP is more recommended for distinguishing species of macrofungi.

The population genetics analysis using AMOVA was also shown to be useful for recognizing species boundaries of macrofungi. Since heterozygotes can be distinguished based on SNP data, AMOVA of SNP data allows for quantifying levels of gene flow among populations, which can provide important clues for recognizing reproductive isolation^54^. Wright's *F*-statistics is commonly used to test deviation from the Hardy–Weinberg equilibrium^26^. Among analyses based on analogs of *F*-statistics, AMOVA is characterized by its flexibility in the use of different hierarchical levels in the analyzed population structure^55^. Such flexibility is beneficial for population genetics studies of macrofungi, for which it is often necessary to collect samples from multiple areas to secure a sufficient number of samples for statistical tests. However, one of the limitations of this method is the necessity of presuming hypothetical species to be tested using AMOVA (e.g., haplotype A and B in the *H. fasciculare* complex), which need to be determined by the dataset independent of the SNP data, such as mitochondrial genes. The second limitation is that the presence/absence of reproductive isolation becomes unconvincing if geographical distributions of hypothetical species do not overlap^24^. Nevertheless, AMOVA for SNP data is an effective and practical tool for detecting reproductive isolation in natural populations.

In summary, our findings indicate that single gene genealogy and comparative gene genealogies may lead to invalid species delimitation among closely related macrofungi. Instead, species boundaries should be distinguished using adequate statistical methods with many loci. Because both EPA-bPTP and AMOVA have some limitations, use of both methods would compensate for each method’s shortcomings and thereby provide reliable results for delimitating cryptic macrofungal species.

## Conclusion

As expected, the species boundaries of the *H. fasciculare* complex remain unclear when analyzed solely by comparative gene genealogies of mt_rRNA and ITS sequences. In contrast, both EPA-bPTP based on the phylogeny of concatenated nuclear sequences and AMOVA based on nuclear SNPs indicate that two mitochondrial haplotypes of the *H. fasciculare* complex represent distinct species, *H. fasciculare* and *H. subviride*. Our findings indicate that caution should be exercised when using single gene genealogy and comparative gene genealogies with few loci for delimiting closely related species of macrofungi. They also suggest that increasing the number of loci used to 20 or more and using both phylogeny-based and population genetics-based statistical frameworks allow for effective and practical macrofungal species delimitation.

## Methods

### Field survey

From June 2014 to November 2016, 95 specimens of the *H. fasciculare* complex were collected from 29 forest sites in Japan (Table S1). One sample of *H. sublateritium* was also collected as an outgroup. Small sections of fruit bodies were removed and stored in 99.5% ethanol for subsequent molecular analysis, and the remaining sections were dried and preserved as voucher specimens. Dried specimens were deposited in the Makino Herbarium of Tokyo Metropolitan University (MAK).

### DNA extraction, PCR amplification, and sequencing

Total DNA was extracted from the tissue of the voucher specimens using a cetyltrimethylammonium bromide (CTAB) method as described previously^35^. Two-step PCR was performed for these samples as described previously^56^. The target regions were the mtLSU and mtSSU, the nuclear ITS region, and 24 single-copy genes (Table 3). For mtSSU and mtLSU, primers were designed as part of the present study. For the other loci, preexisting primers were used^8,56^. After pooling of an equal volume of the respective PCR products, amplicons of 450–600 bp in length were excised and extracted with the E-Gel SizeSelect 2% agarose gel system (Thermo Fisher Scientific). The amplicon libraries were sequenced by 2 × 250 bp paired-end sequencing on a MiSeq platform (Illumina, San Diego, CA, USA) using a MiSeq v2 Reagent NANO Kit according to the manufacturer’s instructions.

### Bioinformatic analyses

Primary data processing of sequence reads was performed using Claident ver. 0.2.2018.05.29^57^ as described previously^56^. The demultiplexed 14,533,614 reads were deposited in the DDBJ Sequence Read Archive (DRA accession: DRA009900). The processed reads were assembled into contigs (unique sequences) in Claident using a similarity cut-off of 100%. The final Claident output files (e.g., "nonchimeras.fasta" and "summary.csv") were further processed using R version 3.3.1^58^. All R scripts used in the molecular analyses are shown in Text S1.

For nuclear loci, unique sequences with a read abundance of ≥ 20% of total 'locus × sample' reads were presumed to represent genotypes and were used for population genetics analysis because it is unlikely that respective genotypes of fruit body samples were represented by only a small fraction of total 'locus × sample' reads. For mitochondrial loci, the most abundant unique sequences were presumed to represent haplotypes.

The unique sequences of each gene were separately aligned with the nucleotide sequences of the same genes of *P. chrysosporium* using MAFFT v7.245^59^ as described previously^56^. Gene sequences that were not detected in more than 50% of the total samples were removed. Alignment data of the remaining genes (FG1021, FG546, FG576, FG652, FG684, FG756, FG761, FG771, FG813, FG848, FG927, FG975, MS320, MS353, MS355, MS358, MS378, MS417, MS444, and MS453, ITS1, ITS2, mtSSU, and mtLSU) were subjected to analyses based on population genetics and molecular phylogenetic inference.

### Molecular phylogenetic inference

The unique sequences of all loci obtained from the same sample were incorporated into the consensus sequence (IUPAC standard) using the "consensus" function of the R package “seqinr ver. 3.4–5”^60^. These consensus sequences (GenBank accession numbers: LC538389-LC540371; Table S2) were used for the subsequent molecular phylogenetic inference. For the ITS1 and ITS2 datasets, regions of ambiguous alignment were removed using Gblocks v0.91b^61^ with options "Allow smaller final blocks" and "Allow gap positions within the final blocks". If the sequences obtained from different samples were identical, those sequences were merged as unique sequences. Molecular phylogenetic inference was performed using the mt_rRNA dataset, ITS dataset, and the nuc_concat dataset (the concatenated sequences of ITS1, ITS2 and 20 single-copy genes), separately (TreeBase ID: S26016). Prior to molecular phylogenetic inference, the nuc_concat dataset was subjected to the congruence among distance matrices^62^ (CADM) test to determine whether the datasets were congruent, using "CADM.global" implemented in the R package "ape ver. 5.1"^63^. Then, the null hypotheses were confirmed to be rejected for all pairwise comparisons (Table S3).

Phylogenetic inference based on the maximum likelihood (ML) method was performed using RAxML ver. 8.1.5^64^, in which the tree searches were repeated 25 times using random sequence addition for generating starting trees. Bootstrap support values were calculated from 1,000 standard bootstrap replications, as implemented in RAxML. Parameters of the GTR Gamma model were estimated separately for each partition according to model selection based on the Akaike information criterion (AIC) using Kakusan 4^65^.

### Phylogeny-based approach to species delimitation using concatenated nuclear sequences

Species delimitation was inferred using the bPTP model, which was integrated with the EPA. Specifically, the ITS1 sequences obtained from the INSD were placed into the ML tree inferred from the nuc_concat dataset using the EPA algorithm as implemented in RAxML. Since the sequencing of the ITS2 was less successful than that of the ITS1 in the present study, the short reads did not include the ITS2. Then, the bPTP model was applied to the phylogenetic tree obtained from the EPA. For bPTP, “*H. sublateritium*” was precluded as an outgroup. The analysis consisted of 1,000,000 Markov Chain Monte Carlo generations, with a thinning every 1,000 generations and a burn-in of 10%.

### Population genetics analysis

Using the "as.matrix.alignment" function of the R package "seqinr", the alignment of each nuclear locus was converted into a matrix of genotypes, where rows and columns represented samples and the nucleotide positions of the DNA sequence, respectively. To reduce biases, samples and loci with many missing datapoints were removed (i.e., 50% cut-off levels). After converting the data frame into a genind object, AMOVA was performed to determine the proportion of nuclear genetic variation that could be attributed to differences between mitochondrial haplotypes, between geographical regions (sampling localities), and between/within samples using the "poppr.amova" function of the R package "poppr ver. 2.8.3"^66^. This test also calculated *Φ* statistics, analogous to Wright's *F*-statistics. The statistical significance of variance components was computed using the Monte Carlo test using the "randtest" function implemented in the R package "ade4"^67^ with 9,999 permutations.

### Observation of morphological characteristics

To compare morphological characteristics, specimens of the *H. fasciculare* complex were examined. The macro- and micromorphological characteristics of basidiomes were described from fresh and dried specimens, respectively. The pilei and stipes of 94 specimens were measured. Microscopic observations were performed under a CX41 optical microscope (OLYMPUS, Tokyo) with material (sections of basidiome tissue) mounted in 5% potassium hydroxide (KOH) solution. Basidiospore measurements were taken at 1,000 × magnification under an optical microscope. The lengths and widths of 5–12 basidiospores were measured for each collection. Between-group differences in pileus and stipe size, spore length and width, and gill cystidia length and width were analyzed based on the Mann–Whitney *U* test using the “wilcox.test” function of R.

## Supplementary information

Supplementary Information

## References

[1] Hawksworth DL (2001). The magnitude of fungal diversity: the 1.5 million species estimate revisited. Mycol. Res..

[2] O'Brien HE, Parrent JL, Jackson JA, Moncalvo JM, Vilgalys R (2005). Fungal community analysis by large-scale sequencing of environmental samples. Appl. Environ. Microbiol..

[3] Hawksworth, D. L. & Lücking, R. In Joseph Heitman *et al.* (eds.) *The Fungal Kingdom* 79–95 (ASM Press, London 2017).

[4] Bickford D (2007). Cryptic species as a window on diversity and conservation. Trends Ecol. Evol..

[5] Crespo A, Lumbsch HT (2010). Cryptic species in lichen-forming fungi. IMA Fungus.

[6] Koufopanou V, Burt A, Szaro T, Taylor JW (2001). Gene genealogies, cryptic species, and molecular evolution in the human pathogen *Coccidioides immitis* and relatives (Ascomycota, Onygenales). Mol. Biol. Evol..

[7] Sato H, Yumoto T, Murakami N (2007). Cryptic species and host specificity in the ectomycorrhizal genus *Strobilomyces* (Strobilomycetaceae). Am J. Bot..

[8] Toju H, Tanabe AS, Yamamoto S, Sato H (2012). High-coverage ITS primers for the DNA-based identification of ascomycetes and basidiomycetes in environmental samples. PLoS ONE.

[9] White, T. J., Bruns, T., Lee, S. & Taylor, J. in M.A. Innis, D.H. Gelfand, J.J. Sninsky, & T.J. White (eds.) *PCR Protocols: A Guide to Methods and Applications*, 315–322 (Academic Press, London 1990).

[10] Schoch CL (2012). Nuclear ribosomal internal transcribed spacer (ITS) region as a universal DNA barcode marker for fungi. Proc. Natl. Acad. Sci..

[11] Nilsson RH, Kristiansson E, Ryberg M, Hallenberg N, Larsson KH (2008). Intraspecific ITS variability in the kingdom Fungi as expressed in the international sequence databases and its implications for molecular species identification. Evol. Bioinform. Online.

[12] Dupuis JR, Roe AD, Sperling FA (2012). Multi-locus species delimitation in closely related animals and fungi: one marker is not enough. Mol. Ecol..

[13] Roe AD, Rice AV, Bromilow SE, Cooke JE, Sperling FA (2010). Multilocus species identification and fungal DNA barcoding: insights from blue stain fungal symbionts of the mountain pine beetle. Mol. Ecol. Resour..

[14] Taylor JW (2000). Phylogenetic species recognition and species concepts in fungi. Fungal Genet. Biol..

[15] Alamouti SM (2011). Gene genealogies reveal cryptic species and host preferences for the pine fungal pathogen *Grosmannia clavigera*. Mol. Ecol..

[16] Kobmoo N, Mongkolsamrit S, Arnamnart N, Luangsa-ard JJ, Giraud T (2019). Population genomics revealed cryptic species within host-specific zombie-ant fungi (*Ophiocordyceps unilateralis*). Mol. Phylogen. Evol..

[17] Koufopanou V, Burt A, Taylor JW (1997). Concordance of gene genealogies reveals reproductive isolation in the pathogenic fungus *Coccidioides immitis*. Proc. Natl. Acad. Sci. USA.

[18] Turissini DA, Gomez OM, Teixeira MM, McEwen JG, Matute DR (2017). Species boundaries in the human pathogen Paracoccidioides. Fungal Genet. Biol..

[19] Kroken S, Taylor JW (2001). A gene genealogical approach to recognize phylogenetic species boundaries in the lichenized fungus Letharia. Mycologia.

[20] Leavitt SD, Esslinger TL, Spribille T, Divakar PK, Lumbsch HT (2013). Multilocus phylogeny of the lichen-forming fungal genus Melanohalea (Parmeliaceae, Ascomycota): insights on diversity, distributions, and a comparison of species tree and concatenated topologies. Mol. Phylogen. Evol..

[21] Parnmen S (2012). Using phylogenetic and coalescent methods to understand the species diversity in the Cladia aggregata complex (Ascomycota, Lecanorales). PLoS ONE.

[22] Kauserud H, Stensrud Ø, Decock C, ShalchianTabrizi K, Schumacher T (2006). Multiple gene genealogies and AFLPs suggest cryptic speciation and long distance dispersal in the basidiomycete *Serpula himantioides* (Boletales). Mol. Ecol..

[23] Sato H, Hattori T (2015). New species of *Boletellus* section *Boletellus* (Boletaceae, Boletales) from Japan, *B. aurocontextus* sp. nov. and *B. areolatus* sp. nov. PLoS ONE.

[24] Fujita MK, Leaché AD, Burbrink FT, McGuire JA, Moritz C (2012). Coalescent-based species delimitation in an integrative taxonomy. Trends Ecol. Evol..

[25] Hudson RR, Coyne JA (2002). Mathematical consequences of the genealogical species concept. Evolution.

[26] Hartl DL, Clark AG, Clark AG (1997). Principles of Population Genetics.

[27] Pons J (2006). Sequence-based species delimitation for the DNA taxonomy of undescribed insects. Syst. Biol..

[28] Reid NM, Carstens BC (2012). Phylogenetic estimation error can decrease the accuracy of species delimitation: a Bayesian implementation of the general mixed Yule-coalescent model. BMC Evol. Biol..

[29] Zhang J, Kapli P, Pavlidis P, Stamatakis A (2013). A general species delimitation method with applications to phylogenetic placements. Bioinformatics.

[30] Berger SA, Krompass D, Stamatakis A (2011). Performance, accuracy, and web server for evolutionary placement of short sequence reads under maximum likelihood. Syst. Biol..

[31] Jackson ND, Carstens BC, Morales AE, O’Meara BC (2017). Species delimitation with gene flow. Syst. Biol..

[32] Douhan GW, Vincenot L, Gryta H, Selosse M-A (2011). Population genetics of ectomycorrhizal fungi: from current knowledge to emerging directions. Fungal Biol..

[33] McDonald BA (1997). The population genetics of fungi: tools and techniques. Phytopathology.

[34] Werth S (2010). Population genetics of lichen-forming fungi—a review. The Lichenologist.

[35] Sato H, Murakami N (2008). Reproductive isolation among cryptic species in the ectomycorrhizal genus *Strobilomyces*: population-level CAPS marker-based genetic analysis. Mol. Phylogen. Evol..

[36] Ammirati JF (1985). Poisonous Mushrooms of the northern United States and Canada.

[37] Imazeki R, Hongo T (1987). Colored Illustrations of Mushrooms of Japan.

[38] Noordeloos M (2011). Strophariaceae sl.

[39] Smith AH (1951). The North American species of naemalotoma. Mycologia.

[40] Bessette AE, Roody WC, Bessette AR, Dunaway DL (2007). Mushrooms of the southeastern United States.

[41] Krieglsteiner GJ (1984). Über neue, seltene, kritische Makromyzeten in der Bundesrepublik Deutschland. V. Zeitschrift für Mykologie.

[42] Seifert KA (2009). Progress towards DNA barcoding of fungi. Mol. Ecol. Resour..

[43] Vialle A (2009). Evaluation of mitochondrial genes as DNA barcode for Basidiomycota. Mol. Ecol. Resour..

[44] Arnold J (1993). Cytonuclear disequilibria in hybrid zones. Annu. Rev. Ecol. Syst..

[45] Avise JC (2000). Phylogeography: The History and Formation of Species.

[46] Hobbie EA, Sánchez FS, Rygiewicz PT (2004). Carbon use, nitrogen use, and isotopic fractionation of ectomycorrhizal and saprotrophic fungi in natural abundance and 13C-labelled cultures. Mycol. Res..

[47] Kohzu A (1999). Natural 13C and 15N abundance of field-collected fungi and their ecological implications. New Phytol..

[48] Kummer, P. *Der Führer in die Pilzkunde : Anleitung zum methodischen, leichten und sichern Bestimmen der in Deutschland vorkommenden Pilze : mit Ausnahme der Schimmel- und allzu winzigen Schleim- und Kern-Pilzchen*. 1–146 (Verlag von E. Luppe's Buchhandlung, 1871).

[49] Berkely MJ, Curtis MA (1869). Fungi Cubenses (Hymenomycetes). J. Linn. Soci..

[50] Miller MR, Dunham JP, Amores A, Cresko WA, Johnson EA (2007). Rapid and cost-effective polymorphism identification and genotyping using restriction site associated DNA (RAD) markers. Genome Res..

[51] Suyama Y, Matsuki Y (2015). MIG-seq: an effective PCR-based method for genome-wide single-nucleotide polymorphism genotyping using the next-generation sequencing platform. Sci. Rep..

[52] Eaton DA (2014). PyRAD: assembly of de novo RADseq loci for phylogenetic analyses. Bioinformatics.

[53] Estoup A, Jarne P, Cornuet JM (2002). Homoplasy and mutation model at microsatellite loci and their consequences for population genetics analysis. Mol. Ecol..

[54] Petit RJ, Excoffier L (2009). Gene flow and species delimitation. Trends Ecol. Evol..

[55] Meirmans PG (2006). Using the AMOVA framework to estimate a standardized genetic differentiation measure. Evolution.

[56] Sato H, Tanabe AS, Toju H (2017). Host shifts enhance diversification of ectomycorrhizal fungi: diversification rate analysis of the ectomycorrhizal fungal genera *Strobilomyces* and *Afroboletus* with an 80-gene phylogeny. New Phytol..

[57] Tanabe AS, Toju H (2013). Two new computational methods for universal DNA barcoding: A benchmark using barcode sequences of bacteria, archaea, animals, fungi, and land plants. PLoS ONE.

[58] R Core Team. *R: A Language and Environment for Statistical Computing. Version 3.3. 1. 2016* (2016).

[59] Katoh K, Misawa K, Kuma K, Miyata T (2002). MAFFT: a novel method for rapid multiple sequence alignment based on fast Fourier transform. Nucl. Acids Res..

[60] Charif D, Lobry JR (2007). SeqinR 10–2: a contributed package to the R project for statistical computing devoted to biological sequences retrieval and analysis. Struct. Approach Seq. Evol..

[61] Castresana J (2000). Selection of conserved blocks from multiple alignments for their use in phylogenetic analysis. Mol. Biol. Evol..

[62] Campbell V, Legendre P, Lapointe F-J (2011). The performance of the congruence among distance matrices (CADM) test in phylogenetic analysis. BMC Evol. Biol..

[63] Paradis E, Claude J, Strimmer K (2004). APE: analyses of phylogenetics and evolution in R language. Bioinformatics.

[64] Stamatakis A (2006). RAxML-VI-HPC: maximum likelihood-based phylogenetic analyses with thousands of taxa and mixed models. Bioinformatics.

[65] Tanabe AS (2011). Kakusan4 and Aminosan: two programs for comparing nonpartitioned, proportional and separate models for combined molecular phylogenetic analyses of multilocus sequence data. Mol. Ecol. Resour..

[66] Kamvar ZN, Tabima JF, Grünwald NJ (2014). Poppr: an R package for genetic analysis of populations with clonal, partially clonal, and/or sexual reproduction. PeerJ.

[67] Dray S, Dufour A-B (2007). The ade4 package: implementing the duality diagram for ecologists. J. Stat. Soft..

