# Supplementary material for: Molecular prospecting for cryptic species of the Hypholoma fasciculare complex: toward the effective and practical delimitation of cryptic macrofungal species
Source: Sci Rep. 2020 Aug 6;10:13224. doi: 10.1038/s41598-020-70166-z (PMC7413530; doi:10.1038/s41598-020-70166-z)
Supplement: Supplementary file 1 — Supplementary Information [file 41598_2020_70166_MOESM1_ESM.pdf]

Molecular prospecting for cryptic species of the *Hypholoma fasciculare* complex: toward the effective and practical delimitation of cryptic macrofungal species

Hirotoshi Sato[1*], Ryoma Ohta[2], Noriaki Murakami[2]

1. Graduate School of Human and Environmental Studies, Kyoto University, Sakyo, Kyoto, 606-8501, Japan

2. Makino Herbarium, Tokyo Metropolitan University, Hachioji, Tokyo 192-0397, Japan

E-mail (corresponding author): h-sato@sys.bot.kyoto-u.ac.jp (HS)

This file contains one Supplementary Text and three Supplementary Tables.

Text. S1. Scripts of R used for the analyses of molecular phylogeny and population genetics.

Table S1 Voucher information of the Hypholoma specimens used in this study.

Table S2. GenBank accession numbers of the consensus sequences used for the molecular phylogeny.

Table S3. Results of congruence among distance (CADM) matrices test for nuclear loci.

Text S1. Scripts of R used for the analyses of molecular phylogeny and population genetics.

```
############################ Load the packages ##################################

library(ape)
library(adegenet)
library(hierfstat)
library(poppr)
library(seqinr)
library(strataG)


########################### Splitting fasta file ##################################

# Define a function to separate a sequence file according to samples and loci
separate.locus <- function(file = "sequence.fasta", csv="summary.csv") {
	seq.name <- list.files(pattern = file)
	FASTA <- read.FASTA(seq.name)
	rename.FASTA <- chartr("-:", "..", names(FASTA))
	names(FASTA) <- rename.FASTA
	CSV <- read.csv(csv, row.names = 1)
	Nams <- apply(CSV, 1, function(x) {
		names(x[x > 0])
	})
	Reads <- apply(CSV, 1, function(x) {
		x[x > 0]
	})
	Len <- lapply(Reads, length)
	seq.name.rep <- paste(rep(names(Len), times = Len), unlist(Reads), sep = "__")
	FASTA2 <- FASTA[unlist(Nams)]
	names(FASTA2) <- seq.name.rep
	split.names <- matrix(unlist(strsplit(seq.name.rep, "__")), nrow = length(seq.name.rep),
		ncol = 3, byrow = T)
	FASTA.split <- lapply(unique(split.names[, 2]), function(x) {
		FASTA2[which(split.names[, 2] == x)]
	})
	names(FASTA.split) <- unique(split.names[, 2])
	file.names <- paste("raw_", unique(split.names[, 2]), ".fasta", sep = "")
	for (i in 1:length(FASTA.split)) {
		write.FASTA(FASTA.split[[i]], file = file.names[i])
	}
}


# Place a fasta file (e.g.m nonchimeras.fasta) and a summary file (rows and columns represent samples and sequence names, respective; e.g., summary.csv) to the working directory of R. Then, execute the above-defined function to split a fasta file according to samples and loci (sample names, locus names and read abundance are shown in sequence titles of output files in this order).
separate.locus(file="nonchimeras.fasta", csv="summary.csv")
```

################### Processing sequence files prior to the subsequent analyses ##################

```
# Define a function to remove the unique sequences with read abundance <20% of total "locus×sample" reads
remove.infreq <- function(file = "raw.+.fas", cutoff = 0.2) {
	seq.names <- list.files(pattern = file)
	FASTA <- lapply(seq.names, read.FASTA)
	NM <- lapply(FASTA, function(Seq) {
		matrix(unlist(strsplit(names(Seq), "__")), ncol = 3, byrow = T)
	})
	NM <- lapply(NM, as.data.frame)
	NM <- lapply(NM, function(x) {
		data.frame(x[, 1], x[, 2], as.numeric(as.character(x[, 3])))
	})
	names(NM) <- seq.names
	sum.sample <- lapply(NM, function(x) {
		tapply(x[, 3], x[, 1], sum)
	})
	names(sum.sample) <- seq.names
	fas <- list()
	for (i in 1:length(seq.names)) {
		fas[[i]] <- FASTA[[i]][which(NM[[i]][, 3] > sum.sample[[i]][NM[[i]][,
			1]] * cutoff)]
		write.FASTA(fas[[i]], file = gsub("raw", "selected", seq.names)[i])
	}
}


# Remove the unique sequences with read abundance <20% of total "locus×sample" reads using the defined function. Place the output files of "separate.locus" function in the working directory.
remove.infreq(cutoff=0.2)
```

```
############ Processing sequence files for the molecular phylogenetic analyses ###############

# Define the function to perform the multiple sequence alignment using MAFFT
Palign.mafft <- function(file = "selected.+.fasta", mRNA = "mRNA.fasta") {
	seq.names <- list.files(pattern = file)
	aligned.names <- gsub("selected", "Paligned", seq.names)
	Seq <- lapply(seq.names, read.FASTA)
	Names <- gsub("selected_", "", seq.names)
	Names <- gsub(".fasta", "", Names)
	names(Seq) <- Names
	mRNA <- read.FASTA(mRNA)
	for (i in 1:length(Seq))
	{
	Seq[[i]] <- append(mRNA[names(Seq)[i]], Seq[[i]])
	}
	aligned.seq <- lapply(Seq, mafft)
	names(aligned.seq) <- aligned.names
	for (i in 1:length(aligned.seq)) {
		write.FASTA(aligned.seq[[i]], file = aligned.names[i])
	}
}

# Define the function to divide the sequence into exon and intron.
div.exon <- function(file = "Paligned.+.fasta") {
	seq.names <- list.files(pattern = file)
	Seq <- lapply(seq.names, read.alignment, format = "fasta")
	Matrix <- lapply(Seq, as.matrix.alignment)
	Matrix.exon <- lapply(Matrix, function(x){as.data.frame(t(x[-1, which(x[1,] != "-")]))})
	Matrix.intron <- lapply(Matrix, function(x){as.data.frame(t(x[-1, which(x[1,] == "-")]))})
	nams.exon <- gsub("Paligned_", "exon_", seq.names)
	nams.intron <- gsub("Paligned_", "intron_", seq.names)
	for (i in 1:length(Matrix)) {
		write.fasta(Matrix.exon[[i]], names = colnames(Matrix.exon[[i]]), file = nams.exon[i])
		write.fasta(Matrix.intron[[i]], names = colnames(Matrix.intron[[i]]), file = nams.intron[i])
	}
}

# Define the function to incorporate the aligned unique sequenes into the consensus sequence (IUPAC standard)
fasta2consensus <- function(exon = "exon.+.fasta", intron = "intron.+.fasta") {
	exon.names <- list.files(pattern = exon)
	intron.names <- list.files(pattern = intron)
	seq.names = c(exon.names, intron.names)
	Seq <- lapply(seq.names, read.alignment, format = "fasta", forceToLower = FALSE)
	make.consensus <- function(Seq) {
		Seq.matrix <- as.matrix.alignment(Seq)
		NM <- matrix(unlist(strsplit(rownames(Seq.matrix), "__")), ncol = 3, byrow = T)
		colnames(NM) <- c("Sample", "Gene", "Read")
		Seq.Matrix <- data.frame(NM[, "Sample"], Seq.matrix)
		colnames(Seq.Matrix)[1] <- "Sample"
		Seq.split <- split(Seq.Matrix, Seq.Matrix$Sample)
		Seq.split <- lapply(Seq.split, function(x) {
			x[, -1]
		})
		factor2character <- function(x) {
			X <- as.data.frame(x)
			sapply(X, as.character)
```

```
			}
			Seq.split <- lapply(Seq.split, factor2character)
			Consensus <- function(x) {
				ConSeq <- seqinr::consensus(x, method = "IUPAC")
				ConSeq[is.na(ConSeq) == 1] = "-"
				names(ConSeq) <- seq(1, length(ConSeq))
				return(ConSeq)
			}
			consensus.matrix <- lapply(Seq.split, function(x) {
				Consensus(matrix(unlist(x), ncol = dim(Seq.matrix)[2]))
			})
			consensus.matrix
		}
		Con.seq <- lapply(Seq, make.consensus)
		nams <- gsub("aligned_", "", seq.names)
		nams <- gsub(".fasta", "", nams)
		names(Con.seq) <- nams
		file.names.c <- mapply(paste, sep = "", rep("consensus_", length(Con.seq)),
			names(Con.seq), rep(".fasta", length(Con.seq)))
		for (i in 1:length(Con.seq)) {
			write.fasta(Con.seq[[i]], names = names(Con.seq[[i]]), file.out = file.names.c[i])
		}
}

# Define the function to create the concatenate sequences of multiple loci
make.concatseq <- function(file = "consensus.+.fasta", cutoff.sample = 0.5, cutoff.locus = 0.5) {
		seq.names <- list.files(pattern = file)
		Con.Seq <- lapply(seq.names, read.alignment, format = "fasta", forceToLower = FALSE)
		Con.seq <- lapply(Con.Seq, as.matrix.alignment)
		Con.seq1 <- lapply(Con.seq, function(x) {
			apply(x, 1, list)
		})
		sample.names <- sort(unique(unlist(sapply(Con.seq, rownames))))
		consensus.seq.all <- function(Seq) {
			Mat <- matrix(nrow = length(sample.names), ncol = length(unlist(Seq))/length(Seq))
			Mat[is.na(Mat) == 1] = "-"
			z <- apply(Mat, 1, list)
			Z <- lapply(z, unlist)
			names(Z) <- sample.names
			Z[names(Seq)] = Seq
			return(Z)
		}
		Con.seq.all <- lapply(Con.seq1, consensus.seq.all)
		Con.S1 <- lapply(Con.seq.all, as.data.frame)
		Con.S2 <- lapply(Con.S1, t)
		Con.S3 <- as.data.frame(Con.S2)
		colnames(Con.S3) <- 1:dim(Con.S3)[2]
		rownames(Con.S3) <- sample.names
		Con.S4 <- apply(Con.S3, 1, list)
		Con.S5 <- lapply(Con.S4, unlist)
		names(Con.S5) <- sample.names
		write.fasta(Con.S5, names = sample.names, file.out = "concatseq.fasta")
		missing.in.sample <- apply(Con.S3, 1, function(x) {
			sum(x == "-")
		})
		missing.in.locus <- apply(Con.S3, 2, function(x) {
			sum(x == "-")
```

```
    })
    Con.S3.ext <- Con.S3[which(missing.in.sample < dim(Con.S3)[2] * cutoff.sample),
        which(missing.in.locus < dim(Con.S3)[1] * cutoff.locus)]
    Con.S4.ext <- apply(Con.S3.ext, 1, list)
    Con.S5.ext <- lapply(Con.S4.ext, unlist)
    sample.names.ext <- sample.names[which(missing.in.sample < dim(Con.S3)[2] *
        cutoff.sample)]
    names(Con.S5.ext) <- sample.names.ext
    write.fasta(Con.S5.ext, names = sample.names.ext, file.out = "concatseq_rm.fasta")
}
```

```
# Perform the multiple alignment using the defined function. Place the output files of "remove.infreq" function in the working directory. Also place the fasta file of mRNA sequences (e.g., mRNA.fasta), for which the sequence title correspond to locus name. The output files are needed to be corrected manually so that the last two nucleotides of introns are GT(GC) and AG.
Palign.mafft()

# Divide the sequence into exon and intron using the defined function.
div.exon()

# Incorporate the aligned unique sequenes into the consensus sequence using the defined function. Place the output files of "div.exon" in the working directory of R.
fasta2consensus()

# Create the concatenate sequences of multiple loci using the defined function. Place the output files of "fasta2consensus" in the working directory of R.
make.concatseq()
```

###################### Population genetics analysis (AMOVA) ################################

```
# Define a function to perform the multiple sequence alignment using MAFFT
align.mafft <- function(file = "selected.+.fasta") {
	seq.names <- list.files(pattern = file)
	aligned.names <- gsub("selected", "aligned", seq.names)
	Seq <- lapply(seq.names, read.FASTA)
	names(Seq) <- seq.names
	aligned.seq <- lapply(Seq, mafft)
	names(aligned.seq) <- aligned.names
	for (i in 1:length(aligned.seq)) {
		write.FASTA(aligned.seq[[i]], file = aligned.names[i])
	}
}

# Define the function to create SNP data from multiple alignment sequences
fasta2genotype <- function(file = "aligned.+.fasta") {
	seq.names <- list.files(pattern = file)
	Seq <- lapply(seq.names, read.alignment, format = "fasta", forceToLower = FALSE)
	convert.genotype <- function(Seq) {
		Seq.matrix <- as.matrix.alignment(Seq)
		NM <- matrix(unlist(strsplit(rownames(Seq.matrix), "__")), ncol = 3, byrow = T)
		colnames(NM) <- c("Sample", "Gene", "Read")
		Seq.Matrix <- data.frame(NM[, "Sample"], Seq.matrix)
		colnames(Seq.Matrix)[1] <- "Sample"
		Seq.split <- split(Seq.Matrix, Seq.Matrix$Sample)
		Seq.split <- lapply(Seq.split, function(x) {
			x[, -1]
		})
		Seq.geno <- lapply(Seq.split, function(x) {
			apply(x, 2, function(x) {
				unique(sort(x))
			})
		})
		make.genotype <- function(x) {
			x <- unlist(x)
			if (length(x) == 1) {
				paste(x, x, sep = "")
			} else {
				paste(x[1], x[2], sep = "")
			}
		}
		genotype <- sapply(Seq.geno, function(x) {
			lapply(x, make.genotype)
		})
		genotype[genotype != "aa" & genotype != "ag" & genotype != "ac" & genotype !=
			"at" & genotype != "cc" & genotype != "cg" & genotype != "ct" & genotype !=
			"gg" & genotype != "gt" & genotype != "tt"] = NA
		rownames(genotype) <- 1:dim(genotype)[1]
		return(t(genotype))
	}
	Genotype <- lapply(Seq, convert.genotype)
	nams <- gsub("aligned_", "", seq.names)
	nams <- gsub(".fasta", "", nams)
	names(Genotype) <- nams
	file.names <- mapply(paste, sep = "", rep("SNP_", length(Genotype)), names(Genotype),
		rep(".csv", length(Genotype)))
```

```
	for (i in 1:length(Genotype)) {
		write.csv(Genotype[[i]], file = file.names[i])
	}
	Sample.names <- sort(unique(unlist(sapply(Genotype, rownames))))
	contain.all.samples <- function(x) {
		Dat <- matrix(nrow = length(Sample.names), ncol = dim(x)[2])
		rownames(Dat) <- Sample.names
		colnames(Dat) <- colnames(x)
		X <- matrix(unlist(x), nrow = dim(x)[1], ncol = dim(x)[2])
		rownames(X) <- rownames(x)
		colnames(X) <- colnames(x)
		Dat[rownames(x), ] = X
		return(Dat)
	}
	Genotype.all <- lapply(Genotype, contain.all.samples)
	SNP <- as.data.frame(Genotype.all)
	RL <- sapply(Genotype.all, dim)[2, ]
	colnames(SNP) <- mapply(paste, sep = "_", rep(names(RL), times = RL),
		unlist(sapply(Genotype.all, colnames)))
	write.csv(SNP, file = "SNP_whole.csv")
}


# Perform the multiple sequence alignment using the defined function. Place the output files of "remove.infreq" function in the working directory.
align.mafft()

# Create SNP data from multiple alignment sequences using the defined function. Place the output files of "align.mafft" in the working directory of R.
fasta2genotype()

# Read SNP data
SNP <- read.csv("SNP_whole.csv", , row.names = 1, stringsAsFactors = F)

# Remove samples and loci with many missing data (50% cut-off levels)
SNP <- remove.na(SNP, cutoff.sample = 0.5)

# Define the hierarchical structure, including hyphothetical species and samplig locality. Prepare the summary file (OTU.csv) depecting the hierarchical structure where the first, second and third columns represent sample names, hypothetical species, and sampling locality, respectively.
OTU <- read.csv("OTU.csv", row.names = 1, stringsAsFactors = F)

# Convert the data frame to "genind" object
SNP.sp <- data.frame(OTU[rownames(SNP), 1], SNP)
colnames(SNP.sp)[1] <- "OTU"
SNP.genind <- df2genind(SNP.sp[, -1], sep = "", ind.names = rownames(SNP.sp), pop = SNP.sp$OTU, ploidy = 2)
strata(SNP.genind) <- OTU[rownames(SNP.sp), ]

# Perform the AMOVA for the SNP data
amova.result <- poppr.amova(SNP.genind, ~OTU/Region, cutoff = 0.5, method = "ade4")

# Compute the statistical significance of variance components with Monte-Carlo test
amova.test <- randtest(amova.result, nrepet = 9999)
```

```
# Create summary of AMOVA results
amova.Results <- amova.result$results
amova.componentsofcovariance <- amova.result$componentsofcovariance
amova.statphi <- c(amova.result$statphi[4:2, 1], "-", "-")
amova.tests <- c(amova.test$pvalue[4:1], "-")
amova.Results <- cbind(c("Between Haplotypes", "Between Regions Within Haplotype", "Between Samples Within Region", "Within Samples", "Total"), amova.Results, amova.componentsofcovariance, amova.statphi, amova.tests)
colnames(amova.Results) <- c("Variaton", "Df", "Sum Sq", "Mean Sq", "Sigma", "%", "phi", "P")
write.csv(amova.Results, file = "amova_results.csv", row.names = FALSE)
```

Table S1 Voucher information of the *Hypholoma* specimens used in this study.

Strain	Species	Locality	Locality ID
F01	H. fasciculare	Tokyo: Bessho, Hachioji	Tok1
F02	H. fasciculare	Tokyo: Naganumamachi, Hachioji	Tok5
F03	H. fasciculare	Tokyo: Bessho, Hachioji	Tok1
F04	H. fasciculare	Gunma: Naegashimamachi, Maebashi	Gun
F05	H. fasciculare	Tokyo: Minamiosawa, Hachioji	Tok4
F06	H. fasciculare	Tokyo: Naganumamachi, Hachioji	Tok5
F07	H. fasciculare	Tokyo: Naganumamachi, Hachioji	Tok5
F08	H. fasciculare	Tokyo: Bessho, Hachioji	Tok10
F09	H. fasciculare	Tokyo: Bessho, Hachioji	Tok1
F10	H. fasciculare	Tokyo: Bessho, Hachioji	Tok1
F11	H. fasciculare	Tokyo: Bessho, Hachioji	Tok1
F12	H. fasciculare	Kanagawa: Midori Ward, Sagamihara	Kan4
F13	H. fasciculare	Kyoto: Yamashina Ward, Kyoto	Kyo
F14	H. fasciculare	Tokyo: Bessho, Hachioji	Tok1
F15	H. fasciculare	Tokyo: Bessho, Hachioji	Tok1
F16	H. fasciculare	Tokyo: Takaomachi, Hachioji	Tok6
F17	H. fasciculare	Kanagawa: Midori Ward, Sagamihara	Kan4
F18	H. fasciculare	Kagoshima: Yakushima, Kumage District	Kag
F19	H. fasciculare	Tokyo: Minamiosawa, Hachioji	Tok4
F20	H. fasciculare	Shiga: Osaka, Otsu	Shi2
F21	H. fasciculare	Shiga: Osaka, Otsu	Shi2
F22	H. fasciculare	Shiga: Osaka, Otsu	Shi2
F23	H. fasciculare	Shiga: Osaka, Otsu	Shi2
F24	H. fasciculare	Shiga: Osaka, Otsu	Shi2
F25	H. fasciculare	Shiga: Jingucho, Otsu	Shi1
F26	H. fasciculare	Tokyo: Bessho, Hachioji	Tok1
F27	H. fasciculare	Tokyo: Bessho, Hachioji	Tok1
F28	H. fasciculare	Kanagawa: Tsuzuki Ward, Yokohama	Kan3
F29	H. fasciculare	Tokyo: Naganumamachi, Hachioji	Tok5
F30	H. fasciculare	Tokyo: Naganumamachi, Hachioji	Tok5
F31	H. fasciculare	Kanagawa: Aoba Ward, Yokohama	Kan1
F32	H. fasciculare	Kanagawa: Midori Ward, Yokohama	Kan2
F33	H. fasciculare	Tokyo: Bessho, Hachioji	Tok1
F34	H. fasciculare	Tokyo: Minamiosawa, Hachioji	Tok4
F35	H. fasciculare	Tokyo: Naganumamachi, Hachioji	Tok9
F36	H. fasciculare	Tokyo: Naganumamachi, Hachioji	Tok5
F37	H. fasciculare	Tokyo: Naganumamachi, Hachioji	Tok5
F38	H. fasciculare	Tokyo: Yanokuchi, Inagi	Tok8
F39	H. fasciculare	Tokyo: Yanokuchi, Inagi	Tok8
F40	H. fasciculare	Tokyo: Kinutakoen, Setagaya	Tok3
F41	H. fasciculare	Tokyo: Kinutakoen, Setagaya	Tok3
F42	H. fasciculare	Oita: Yufuincho Kawakami, Yufu	Oit
F43	H. fasciculare	Oita: Yufuincho Kawakami, Yufu	Oit
F44	H. fasciculare	Kanagawa: Tsuzuki Ward, Yokohama	Kan3
F45	H. fasciculare	Yamanashi: Sasagomachi, Otsuki	Yam2
F46	H. fasciculare	Tochigi: Chugushi, Nikko	Toc
F47	H. fasciculare	Tochigi: Chugushi, Nikko	Toc
F48	H. fasciculare	Tochigi: Chugushi, Nikko	Toc
F49	H. fasciculare	Tochigi: Chugushi, Nikko	Toc
F50	H. fasciculare	Tochigi: Chugushi, Nikko	Toc
F51	H. fasciculare	Kanagawa: Midori Ward, Yokohama	Kan2

F52	H. fasciculare	Kanagawa: Midori Ward, Sagamihara	Kan5
F54	H. fasciculare	Tokyo: Tangimachi, Hachioji	Tok7
F55	H. fasciculare	Tokyo: Bessho, Hachioji	Tok1
F56	H. fasciculare	Tokyo: Bessho, Hachioji	Tok1
F57	H. fasciculare	Tokyo: Bessho, Hachioji	Tok1
F58	H. fasciculare	Tokyo: Bessho, Hachioji	Tok1
F59	H. fasciculare	Tokyo: Higashiyamato, Narahashi	Tok2
F60	H. fasciculare	Tokyo: Higashiyamato, Narahashi	Tok2
F61	H. fasciculare	Shiga: Setaoecho, Otsu	Shi3
F62	H. fasciculare	Shiga: Setaoecho, Otsu	Shi3
F63	H. fasciculare	Yamanashi: Sasagomachi, Otsuki	Yam2
F64	H. fasciculare	Yamanashi: Sasagomachi, Otsuki	Yam2
F65	H. fasciculare	Yamanashi: Sasagomachi, Otsuki	Yam2
F66	H. fasciculare	Tokyo: Bessho, Hachioji	Tok1
F67	H. fasciculare	Tokyo: Bessho, Hachioji	Tok1
F68	H. fasciculare	Tokyo: Bessho, Hachioji	Tok1
F69	H. fasciculare	Tokyo: Bessho, Hachioji	Tok1
F70	H. fasciculare	Tokyo: Bessho, Hachioji	Tok1
F71	H. fasciculare	Tokyo: Tangimachi, Hachioji	Tok7
F72	H. fasciculare	Tokyo: Tangimachi, Hachioji	Tok7
F73	H. fasciculare	Tokyo: Tangimachi, Hachioji	Tok7
F74	H. fasciculare	Chiba: Sakae, Inba District	Chi
F75	H. fasciculare	Chiba: Sakae, Inba District	Chi
F76	H. fasciculare	Chiba: Sakae, Inba District	Chi
F77	H. fasciculare	Chiba: Sakae, Inba District	Chi
F78	H. fasciculare	Chiba: Sakae, Inba District	Chi
F79	H. fasciculare	Chiba: Sakae, Inba District	Chi
F80	H. fasciculare	Yamanashi: Magi, Otsukimachi, Ootsuki	Yam1
F81	H. fasciculare	Yamanashi: Magi, Otsukimachi, Ootsuki	Yam1
F82	H. fasciculare	Yamanashi: Magi, Otsukimachi, Ootsuki	Yam1
F83	H. fasciculare	Yamanashi: Magi, Otsukimachi, Ootsuki	Yam1
F84	H. fasciculare	Yamanashi: Magi, Otsukimachi, Ootsuki	Yam1
F85	H. fasciculare	Yamanashi: Tsurushima, Uenohara, Ootsuki	Yam3
F86	H. fasciculare	Nagano: iiyama	Nag
F87	H. fasciculare	Nagano: iiyama	Nag
F88	H. fasciculare	Nagano: iiyama	Nag
F89	H. fasciculare	Nagano: iiyama	Nag
F90	H. fasciculare	Yamagata: Oguni, Nishiokitama District	Yamg
F91	H. fasciculare	Yamagata: Oguni, Nishiokitama District	Yamg
F92	H. fasciculare	Yamagata: Oguni, Nishiokitama District	Yamg
F93	H. fasciculare	Yamagata: Oguni, Nishiokitama District	Yamg
F94	H. fasciculare	Yamanashi: Tsurushima, Uenohara, Ootsuki	Yam3
F95	H. fasciculare	Tokyo: Naganumamachi, Hachioji	Tok5
yk334	H. fasciculare	Kagoshima: Yakushima, Kumage District	Kag
FS01	H. sublateritium	Kyoto: Miyamacho, Nantan	

Table S2. GenBank accession numbers of the consensus sequences used for the molecular phylogeny.

Strain	GenBank Accession (locus)
F01	LC538389(FG546), LC538483(FG576), LC538578(FG652), LC538721(FG756), LC538812(FG761), LC538897(FG771), LC538992(FG813), LC539083(FG848), LC539178(FG927), LC539274(FG975), LC539409(MS320), LC539505(MS353), LC539558(MS355), LC539635(MS358), LC539813(MS444), LC539905(MS453), LC540180(mtLSU), LC540276(mtSSU)
F02	LC538390(FG546), LC538484(FG576), LC538722(FG756), LC538813(FG761), LC538898(FG771), LC538993(FG813), LC539084(FG848), LC539179(FG927), LC539275(FG975), LC539410(MS320), LC539506(MS353), LC539559(MS355), LC539636(MS358), LC539814(MS444), LC539906(MS453), LC540181(mtLSU), LC540277(mtSSU)
F03	LC538391(FG546), LC538485(FG576), LC538723(FG756), LC538814(FG761), LC538899(FG771), LC538994(FG813), LC539085(FG848), LC539180(FG927), LC539276(FG975), LC539411(MS320), LC539507(MS353), LC539560(MS355), LC539637(MS358), LC539728(MS378), LC539815(MS444), LC539907(MS453), LC540182(mtLSU), LC540278(mtSSU)
F04	LC538392(FG546), LC538486(FG576), LC538579(FG652), LC538647(FG684), LC538724(FG756), LC538815(FG761), LC538900(FG771), LC538995(FG813), LC539086(FG848), LC539181(FG927), LC539277(FG975), LC539412(MS320), LC539508(MS353), LC539561(MS355), LC539638(MS358), LC539729(MS378), LC539778(MS417), LC539816(MS444), LC539908(MS453), LC540183(mtLSU), LC540279(mtSSU)
F05	LC538393(FG546), LC538487(FG576), LC538648(FG684), LC538725(FG756), LC538816(FG761), LC538901(FG771), LC538996(FG813), LC539087(FG848), LC539182(FG927), LC539278(FG975), LC539413(MS320), LC539509(MS353), LC539562(MS355), LC539639(MS358), LC539730(MS378), LC539779(MS417), LC539817(MS444), LC539909(MS453), LC540000(ITS1), LC540091(ITS2), LC540184(mtLSU), LC540280(mtSSU)
F06	LC538394(FG546), LC538488(FG576), LC538580(FG652), LC538649(FG684), LC538726(FG756), LC538817(FG761), LC538902(FG771), LC538997(FG813), LC539088(FG848), LC539183(FG927), LC539279(FG975), LC539414(MS320), LC539510(MS353), LC539563(MS355), LC539640(MS358), LC539731(MS378), LC539818(MS444), LC539910(MS453), LC540001(ITS1), LC540092(ITS2), LC540185(mtLSU), LC540281(mtSSU)
F07	LC538395(FG546), LC538489(FG576), LC538581(FG652), LC538650(FG684), LC538727(FG756), LC538818(FG761), LC538903(FG771), LC538998(FG813), LC539089(FG848), LC539184(FG927), LC539280(FG975), LC539415(MS320), LC539564(MS355), LC539641(MS358), LC539819(MS444), LC539911(MS453), LC540002(ITS1), LC540093(ITS2), LC540186(mtLSU), LC540282(mtSSU)
F08	LC538396(FG546), LC538490(FG576), LC538582(FG652), LC538651(FG684), LC538728(FG756), LC538819(FG761), LC538904(FG771), LC538999(FG813), LC539090(FG848), LC539185(FG927), LC539281(FG975), LC539370(FG1021), LC539416(MS320), LC539511(MS353), LC539565(MS355), LC539642(MS358), LC539780(MS417), LC539820(MS444), LC539912(MS453), LC540003(ITS1), LC540094(ITS2), LC540187(mtLSU), LC540283(mtSSU)
F09	LC538397(FG546), LC538491(FG576), LC538583(FG652), LC538652(FG684), LC538729(FG756), LC538820(FG761), LC538905(FG771), LC539000(FG813), LC539091(FG848), LC539186(FG927), LC539282(FG975), LC539371(FG1021), LC539417(MS320), LC539512(MS353), LC539566(MS355), LC539643(MS358), LC539821(MS444), LC539913(MS453), LC540004(ITS1), LC540095(ITS2), LC540188(mtLSU), LC540284(mtSSU)

F10	LC538398(FG546), LC538492(FG576), LC538584(FG652), LC538653(FG684), LC538730(FG756), LC538821(FG761), LC538906(FG771), LC539001(FG813), LC539092(FG848), LC539187(FG927), LC539283(FG975), LC539418(MS320), LC539513(MS353), LC539567(MS355), LC539644(MS358), LC539732(MS378), LC539781(MS417), LC539822(MS444), LC539914(MS453), LC540005(ITS1), LC540096(ITS2), LC540189(mtLSU), LC540285(mtSSU)
F11	LC538399(FG546), LC538493(FG576), LC538585(FG652), LC538654(FG684), LC538731(FG756), LC538907(FG771), LC539002(FG813), LC539093(FG848), LC539188(FG927), LC539284(FG975), LC539419(MS320), LC539514(MS353), LC539568(MS355), LC539645(MS358), LC539733(MS378), LC539782(MS417), LC539823(MS444), LC539915(MS453), LC540006(ITS1), LC540097(ITS2), LC540190(mtLSU), LC540286(mtSSU)
F12	LC538400(FG546), LC538494(FG576), LC538655(FG684), LC538732(FG756), LC538822(FG761), LC538908(FG771), LC539003(FG813), LC539094(FG848), LC539189(FG927), LC539285(FG975), LC539420(MS320), LC539515(MS353), LC539569(MS355), LC539646(MS358), LC539734(MS378), LC539824(MS444), LC539916(MS453), LC540007(ITS1), LC540098(ITS2), LC540191(mtLSU), LC540287(mtSSU)
F13	LC538401(FG546), LC538495(FG576), LC538586(FG652), LC538656(FG684), LC538733(FG756), LC538823(FG761), LC538909(FG771), LC539004(FG813), LC539095(FG848), LC539190(FG927), LC539286(FG975), LC539421(MS320), LC539570(MS355), LC539647(MS358), LC539825(MS444), LC539917(MS453), LC540008(ITS1), LC540192(mtLSU), LC540288(mtSSU)
F14	LC538402(FG546), LC538496(FG576), LC538587(FG652), LC538657(FG684), LC538734(FG756), LC538824(FG761), LC538910(FG771), LC539005(FG813), LC539096(FG848), LC539191(FG927), LC539287(FG975), LC539422(MS320), LC539516(MS353), LC539571(MS355), LC539648(MS358), LC539735(MS378), LC539783(MS417), LC539826(MS444), LC539918(MS453), LC540009(ITS1), LC540099(ITS2), LC540193(mtLSU), LC540289(mtSSU)
F15	LC538403(FG546), LC538497(FG576), LC538588(FG652), LC538658(FG684), LC538735(FG756), LC538825(FG761), LC538911(FG771), LC539006(FG813), LC539097(FG848), LC539192(FG927), LC539288(FG975), LC539423(MS320), LC539517(MS353), LC539572(MS355), LC539649(MS358), LC539736(MS378), LC539827(MS444), LC539919(MS453), LC540010(ITS1), LC540100(ITS2), LC540194(mtLSU), LC540290(mtSSU)
F16	LC538404(FG546), LC538498(FG576), LC538736(FG756), LC538826(FG761), LC538912(FG771), LC539007(FG813), LC539098(FG848), LC539193(FG927), LC539289(FG975), LC539372(FG1021), LC539424(MS320), LC539518(MS353), LC539573(MS355), LC539650(MS358), LC539737(MS378), LC539828(MS444), LC539920(MS453), LC540011(ITS1), LC540101(ITS2), LC540195(mtLSU), LC540291(mtSSU)
F17	LC538405(FG546), LC538499(FG576), LC538737(FG756), LC538913(FG771), LC539008(FG813), LC539099(FG848), LC539194(FG927), LC539290(FG975), LC539425(MS320), LC539519(MS353), LC539574(MS355), LC539651(MS358), LC539829(MS444), LC539921(MS453), LC540012(ITS1), LC540102(ITS2), LC540196(mtLSU), LC540292(mtSSU)
F18	LC538406(FG546), LC538500(FG576), LC538659(FG684), LC538738(FG756), LC538827(FG761), LC538914(FG771), LC539009(FG813), LC539100(FG848), LC539195(FG927), LC539291(FG975), LC539426(MS320), LC539520(MS353), LC539575(MS355), LC539652(MS358), LC539784(MS417), LC539830(MS444), LC539922(MS453), LC540013(ITS1), LC540103(ITS2), LC540197(mtLSU), LC540293(mtSSU)
F19	LC538407(FG546), LC538501(FG576), LC538589(FG652), LC538660(FG684), LC538739(FG756), LC538828(FG761), LC538915(FG771), LC539010(FG813), LC539101(FG848), LC539196(FG927), LC539292(FG975), LC539427(MS320), LC539521(MS353), LC539576(MS355), LC539653(MS358), LC539831(MS444),

	LC539923(MS453), LC540014(ITS1), LC540104(ITS2), LC540198(mtLSU), LC540294(mtSSU)
F20	LC538408(FG546), LC538502(FG576), LC538590(FG652), LC538740(FG756), LC538829(FG761), LC538916(FG771), LC539011(FG813), LC539102(FG848), LC539197(FG927), LC539293(FG975), LC539428(MS320), LC539522(MS353), LC539577(MS355), LC539654(MS358), LC539738(MS378), LC539832(MS444), LC539924(MS453), LC540015(ITS1), LC540105(ITS2), LC540199(mtLSU), LC540295(mtSSU)
F21	LC538409(FG546), LC538503(FG576), LC538591(FG652), LC538661(FG684), LC538741(FG756), LC538830(FG761), LC538917(FG771), LC539012(FG813), LC539103(FG848), LC539198(FG927), LC539294(FG975), LC539429(MS320), LC539523(MS353), LC539578(MS355), LC539655(MS358), LC539739(MS378), LC539785(MS417), LC539833(MS444), LC539925(MS453), LC540016(ITS1), LC540106(ITS2), LC540200(mtLSU), LC540296(mtSSU)
F22	LC538410(FG546), LC538504(FG576), LC538592(FG652), LC538662(FG684), LC538742(FG756), LC538831(FG761), LC538918(FG771), LC539013(FG813), LC539104(FG848), LC539199(FG927), LC539295(FG975), LC539373(FG1021), LC539430(MS320), LC539524(MS353), LC539579(MS355), LC539656(MS358), LC539740(MS378), LC539834(MS444), LC539926(MS453), LC540017(ITS1), LC540107(ITS2), LC540201(mtLSU), LC540297(mtSSU)
F23	LC538411(FG546), LC538505(FG576), LC538743(FG756), LC538832(FG761), LC538919(FG771), LC539014(FG813), LC539105(FG848), LC539200(FG927), LC539296(FG975), LC539374(FG1021), LC539431(MS320), LC539657(MS358), LC539741(MS378), LC539786(MS417), LC539835(MS444), LC539927(MS453), LC540018(ITS1), LC540108(ITS2), LC540202(mtLSU), LC540298(mtSSU)
F24	LC538412(FG546), LC538506(FG576), LC538663(FG684), LC538744(FG756), LC538833(FG761), LC538920(FG771), LC539106(FG848), LC539201(FG927), LC539297(FG975), LC539375(FG1021), LC539432(MS320), LC539580(MS355), LC539658(MS358), LC539836(MS444), LC539928(MS453), LC540019(ITS1), LC540109(ITS2), LC540203(mtLSU), LC540299(mtSSU)
F25	LC538413(FG546), LC538507(FG576), LC538593(FG652), LC538664(FG684), LC538745(FG756), LC538921(FG771), LC539015(FG813), LC539107(FG848), LC539202(FG927), LC539298(FG975), LC539376(FG1021), LC539433(MS320), LC539525(MS353), LC539581(MS355), LC539659(MS358), LC539742(MS378), LC539787(MS417), LC539837(MS444), LC539929(MS453), LC540020(ITS1), LC540110(ITS2), LC540204(mtLSU), LC540300(mtSSU)
F26	LC538414(FG546), LC538508(FG576), LC538594(FG652), LC538665(FG684), LC538746(FG756), LC538834(FG761), LC538922(FG771), LC539016(FG813), LC539108(FG848), LC539203(FG927), LC539299(FG975), LC539434(MS320), LC539526(MS353), LC539582(MS355), LC539660(MS358), LC539838(MS444), LC539930(MS453), LC540021(ITS1), LC540111(ITS2), LC540205(mtLSU), LC540301(mtSSU)
F27	LC538415(FG546), LC538509(FG576), LC538595(FG652), LC538666(FG684), LC538747(FG756), LC538835(FG761), LC538923(FG771), LC539017(FG813), LC539109(FG848), LC539204(FG927), LC539300(FG975), LC539435(MS320), LC539527(MS353), LC539583(MS355), LC539661(MS358), LC539743(MS378), LC539839(MS444), LC539931(MS453), LC540022(ITS1), LC540112(ITS2), LC540206(mtLSU), LC540302(mtSSU)
F28	LC538416(FG546), LC538510(FG576), LC538596(FG652), LC538667(FG684), LC538748(FG756), LC538836(FG761), LC538924(FG771), LC539018(FG813), LC539110(FG848), LC539205(FG927), LC539301(FG975), LC539377(FG1021), LC539436(MS320), LC539528(MS353), LC539584(MS355), LC539662(MS358), LC539744(MS378), LC539840(MS444), LC539932(MS453), LC540023(ITS1), LC540113(ITS2), LC540207(mtLSU), LC540303(mtSSU)

F29	LC538417(FG546), LC538511(FG576), LC538597(FG652), LC538668(FG684), LC538749(FG756), LC538837(FG761), LC538925(FG771), LC539019(FG813), LC539111(FG848), LC539206(FG927), LC539302(FG975), LC539437(MS320), LC539529(MS353), LC539585(MS355), LC539663(MS358), LC539745(MS378), LC539841(MS444), LC539933(MS453), LC540024(ITS1), LC540114(ITS2), LC540208(mtLSU), LC540304(mtSSU)
F30	LC538418(FG546), LC538512(FG576), LC538598(FG652), LC538669(FG684), LC538750(FG756), LC538838(FG761), LC538926(FG771), LC539020(FG813), LC539112(FG848), LC539207(FG927), LC539303(FG975), LC539438(MS320), LC539530(MS353), LC539586(MS355), LC539664(MS358), LC539842(MS444), LC539934(MS453), LC540025(ITS1), LC540115(ITS2), LC540209(mtLSU), LC540305(mtSSU)
F31	LC538419(FG546), LC538513(FG576), LC538599(FG652), LC538670(FG684), LC538751(FG756), LC538839(FG761), LC538927(FG771), LC539021(FG813), LC539113(FG848), LC539208(FG927), LC539304(FG975), LC539378(FG1021), LC539439(MS320), LC539531(MS353), LC539587(MS355), LC539665(MS358), LC539746(MS378), LC539843(MS444), LC539935(MS453), LC540026(ITS1), LC540116(ITS2), LC540210(mtLSU), LC540306(mtSSU)
F32	LC538420(FG546), LC538514(FG576), LC538671(FG684), LC538752(FG756), LC538840(FG761), LC538928(FG771), LC539022(FG813), LC539114(FG848), LC539209(FG927), LC539305(FG975), LC539379(FG1021), LC539440(MS320), LC539588(MS355), LC539666(MS358), LC539788(MS417), LC539844(MS444), LC539936(MS453), LC540027(ITS1), LC540117(ITS2), LC540211(mtLSU), LC540307(mtSSU)
F33	LC538421(FG546), LC538515(FG576), LC538600(FG652), LC538672(FG684), LC538753(FG756), LC538841(FG761), LC538929(FG771), LC539023(FG813), LC539115(FG848), LC539210(FG927), LC539306(FG975), LC539441(MS320), LC539589(MS355), LC539667(MS358), LC539845(MS444), LC539937(MS453), LC540028(ITS1), LC540118(ITS2), LC540212(mtLSU), LC540308(mtSSU)
F34	LC538422(FG546), LC538516(FG576), LC538601(FG652), LC538754(FG756), LC538842(FG761), LC538930(FG771), LC539024(FG813), LC539116(FG848), LC539211(FG927), LC539307(FG975), LC539442(MS320), LC539668(MS358), LC539846(MS444), LC539938(MS453), LC540029(ITS1), LC540119(ITS2), LC540213(mtLSU), LC540309(mtSSU)
F35	LC538423(FG546), LC538517(FG576), LC538602(FG652), LC538673(FG684), LC538755(FG756), LC538843(FG761), LC538931(FG771), LC539025(FG813), LC539117(FG848), LC539212(FG927), LC539308(FG975), LC539443(MS320), LC539669(MS358), LC539847(MS444), LC539939(MS453), LC540030(ITS1), LC540120(ITS2), LC540214(mtLSU), LC540310(mtSSU)
F36	LC538424(FG546), LC538518(FG576), LC538756(FG756), LC538844(FG761), LC538932(FG771), LC539026(FG813), LC539118(FG848), LC539213(FG927), LC539309(FG975), LC539380(FG1021), LC539444(MS320), LC539670(MS358), LC539848(MS444), LC539940(MS453), LC540031(ITS1), LC540121(ITS2), LC540215(mtLSU), LC540311(mtSSU)
F37	LC538425(FG546), LC538519(FG576), LC538603(FG652), LC538674(FG684), LC538757(FG756), LC538845(FG761), LC538933(FG771), LC539027(FG813), LC539119(FG848), LC539214(FG927), LC539310(FG975), LC539381(FG1021), LC539445(MS320), LC539532(MS353), LC539590(MS355), LC539671(MS358), LC539849(MS444), LC539941(MS453), LC540032(ITS1), LC540122(ITS2), LC540216(mtLSU), LC540312(mtSSU)
F38	LC538426(FG546), LC538520(FG576), LC538604(FG652), LC538675(FG684), LC538758(FG756), LC538846(FG761), LC538934(FG771), LC539028(FG813), LC539120(FG848), LC539215(FG927), LC539311(FG975), LC539446(MS320), LC539591(MS355), LC539672(MS358), LC539789(MS417), LC539850(MS444), LC539942(MS453), LC540033(ITS1), LC540123(ITS2), LC540217(mtLSU), LC540313(mtSSU)

F39	LC538427(FG546), LC538521(FG576), LC538605(FG652), LC538676(FG684), LC538759(FG756), LC538935(FG771), LC539029(FG813), LC539121(FG848), LC539216(FG927), LC539312(FG975), LC539447(MS320), LC539533(MS353), LC539592(MS355), LC539673(MS358), LC539790(MS417), LC539851(MS444), LC539943(MS453), LC540034(ITS1), LC540124(ITS2), LC540218(mtLSU), LC540314(mtSSU)
F40	LC538428(FG546), LC538522(FG576), LC538606(FG652), LC538677(FG684), LC538760(FG756), LC538847(FG761), LC538936(FG771), LC539030(FG813), LC539122(FG848), LC539217(FG927), LC539313(FG975), LC539382(FG1021), LC539448(MS320), LC539593(MS355), LC539674(MS358), LC539747(MS378), LC539791(MS417), LC539852(MS444), LC539944(MS453), LC540035(ITS1), LC540125(ITS2), LC540219(mtLSU), LC540315(mtSSU)
F41	LC538429(FG546), LC538523(FG576), LC538607(FG652), LC538678(FG684), LC538761(FG756), LC538848(FG761), LC538937(FG771), LC539031(FG813), LC539123(FG848), LC539218(FG927), LC539314(FG975), LC539383(FG1021), LC539449(MS320), LC539534(MS353), LC539594(MS355), LC539675(MS358), LC539748(MS378), LC539792(MS417), LC539853(MS444), LC539945(MS453), LC540036(ITS1), LC540126(ITS2), LC540220(mtLSU), LC540316(mtSSU)
F42	LC538430(FG546), LC538524(FG576), LC538608(FG652), LC538762(FG756), LC538849(FG761), LC538938(FG771), LC539032(FG813), LC539124(FG848), LC539219(FG927), LC539315(FG975), LC539450(MS320), LC539535(MS353), LC539595(MS355), LC539676(MS358), LC539749(MS378), LC539793(MS417), LC539854(MS444), LC539946(MS453), LC540037(ITS1), LC540127(ITS2), LC540221(mtLSU), LC540317(mtSSU)
F43	LC538431(FG546), LC538525(FG576), LC538609(FG652), LC538679(FG684), LC538763(FG756), LC538850(FG761), LC538939(FG771), LC539033(FG813), LC539125(FG848), LC539220(FG927), LC539316(FG975), LC539451(MS320), LC539536(MS353), LC539596(MS355), LC539677(MS358), LC539750(MS378), LC539855(MS444), LC539947(MS453), LC540038(ITS1), LC540128(ITS2), LC540222(mtLSU), LC540318(mtSSU)
F44	LC538432(FG546), LC538526(FG576), LC538764(FG756), LC538851(FG761), LC538940(FG771), LC539034(FG813), LC539126(FG848), LC539221(FG927), LC539317(FG975), LC539452(MS320), LC539678(MS358), LC539856(MS444), LC539948(MS453), LC540039(ITS1), LC540129(ITS2), LC540223(mtLSU), LC540319(mtSSU)
F45	LC538433(FG546), LC538527(FG576), LC538610(FG652), LC538680(FG684), LC538765(FG756), LC538852(FG761), LC538941(FG771), LC539035(FG813), LC539127(FG848), LC539222(FG927), LC539318(FG975), LC539453(MS320), LC539679(MS358), LC539794(MS417), LC539857(MS444), LC539949(MS453), LC540040(ITS1), LC540130(ITS2), LC540224(mtLSU), LC540320(mtSSU)
F46	LC538434(FG546), LC538528(FG576), LC538611(FG652), LC538766(FG756), LC538853(FG761), LC538942(FG771), LC539036(FG813), LC539128(FG848), LC539223(FG927), LC539319(FG975), LC539454(MS320), LC539597(MS355), LC539680(MS358), LC539858(MS444), LC539950(MS453), LC540041(ITS1), LC540131(ITS2), LC540225(mtLSU), LC540321(mtSSU)
F47	LC538435(FG546), LC538529(FG576), LC538612(FG652), LC538681(FG684), LC538767(FG756), LC538854(FG761), LC538943(FG771), LC539037(FG813), LC539129(FG848), LC539224(FG927), LC539320(FG975), LC539384(FG1021), LC539455(MS320), LC539537(MS353), LC539598(MS355), LC539681(MS358), LC539859(MS444), LC539951(MS453), LC540042(ITS1), LC540132(ITS2), LC540226(mtLSU), LC540322(mtSSU)
F48	LC538436(FG546), LC538530(FG576), LC538768(FG756), LC538855(FG761), LC538944(FG771), LC539038(FG813), LC539130(FG848), LC539225(FG927), LC539321(FG975), LC539456(MS320), LC539599(MS355), LC539682(MS358), LC539751(MS378), LC539860(MS444), LC539952(MS453), LC540043(ITS1), LC540133(ITS2), LC540227(mtLSU), LC540323(mtSSU)

F49	LC538437(FG546), LC538531(FG576), LC538682(FG684), LC538769(FG756), LC538856(FG761), LC538945(FG771), LC539039(FG813), LC539131(FG848), LC539226(FG927), LC539322(FG975), LC539385(FG1021), LC539457(MS320), LC539683(MS358), LC539861(MS444), LC539953(MS453), LC540044(ITS1), LC540134(ITS2), LC540228(mtLSU), LC540324(mtSSU)
F50	LC538438(FG546), LC538532(FG576), LC538946(FG771), LC539132(FG848), LC539227(FG927), LC539323(FG975), LC539458(MS320), LC539600(MS355), LC539954(MS453), LC540045(ITS1), LC540135(ITS2), LC540229(mtLSU), LC540325(mtSSU)
F51	LC538439(FG546), LC538533(FG576), LC538613(FG652), LC538683(FG684), LC538770(FG756), LC538857(FG761), LC538947(FG771), LC539040(FG813), LC539133(FG848), LC539228(FG927), LC539324(FG975), LC539386(FG1021), LC539459(MS320), LC539538(MS353), LC539601(MS355), LC539684(MS358), LC539752(MS378), LC539862(MS444), LC539955(MS453), LC540046(ITS1), LC540136(ITS2), LC540230(mtLSU), LC540326(mtSSU)
F52	LC538440(FG546), LC538534(FG576), LC538614(FG652), LC538771(FG756), LC538858(FG761), LC538948(FG771), LC539041(FG813), LC539134(FG848), LC539229(FG927), LC539325(FG975), LC539460(MS320), LC539602(MS355), LC539685(MS358), LC539863(MS444), LC539956(MS453), LC540047(ITS1), LC540137(ITS2), LC540231(mtLSU), LC540327(mtSSU)
F54	LC538441(FG546), LC538535(FG576), LC538615(FG652), LC538684(FG684), LC538772(FG756), LC538859(FG761), LC538949(FG771), LC539042(FG813), LC539135(FG848), LC539230(FG927), LC539326(FG975), LC539461(MS320), LC539539(MS353), LC539603(MS355), LC539686(MS358), LC539753(MS378), LC539795(MS417), LC539864(MS444), LC539957(MS453), LC540048(ITS1), LC540138(ITS2), LC540232(mtLSU), LC540328(mtSSU)
F55	LC538442(FG546), LC538536(FG576), LC538685(FG684), LC538773(FG756), LC538950(FG771), LC539043(FG813), LC539136(FG848), LC539231(FG927), LC539327(FG975), LC539387(FG1021), LC539462(MS320), LC539540(MS353), LC539604(MS355), LC539687(MS358), LC539865(MS444), LC539958(MS453), LC540049(ITS1), LC540139(ITS2), LC540233(mtLSU), LC540329(mtSSU)
F56	LC538443(FG546), LC538537(FG576), LC538686(FG684), LC538774(FG756), LC538860(FG761), LC538951(FG771), LC539044(FG813), LC539137(FG848), LC539232(FG927), LC539328(FG975), LC539388(FG1021), LC539463(MS320), LC539688(MS358), LC539866(MS444), LC539959(MS453), LC540050(ITS1), LC540140(ITS2), LC540234(mtLSU), LC540330(mtSSU)
F57	LC538444(FG546), LC538538(FG576), LC538775(FG756), LC538861(FG761), LC538952(FG771), LC539045(FG813), LC539138(FG848), LC539233(FG927), LC539329(FG975), LC539389(FG1021), LC539464(MS320), LC539605(MS355), LC539689(MS358), LC539960(MS453), LC540051(ITS1), LC540141(ITS2), LC540235(mtLSU), LC540331(mtSSU)
F58	LC538445(FG546), LC538539(FG576), LC538776(FG756), LC538862(FG761), LC538953(FG771), LC539046(FG813), LC539139(FG848), LC539234(FG927), LC539330(FG975), LC539465(MS320), LC539606(MS355), LC539690(MS358), LC539867(MS444), LC539961(MS453), LC540052(ITS1), LC540142(ITS2), LC540236(mtLSU), LC540332(mtSSU)
F59	LC538446(FG546), LC538540(FG576), LC538616(FG652), LC538687(FG684), LC538777(FG756), LC538863(FG761), LC538954(FG771), LC539047(FG813), LC539140(FG848), LC539235(FG927), LC539331(FG975), LC539390(FG1021), LC539466(MS320), LC539541(MS353), LC539607(MS355), LC539691(MS358), LC539754(MS378), LC539868(MS444), LC539962(MS453), LC540053(ITS1), LC540143(ITS2), LC540237(mtLSU), LC540333(mtSSU)
F60	LC538447(FG546), LC538541(FG576), LC538617(FG652), LC538688(FG684), LC538778(FG756), LC538864(FG761), LC538955(FG771), LC539048(FG813), LC539141(FG848), LC539236(FG927), LC539332(FG975), LC539391(FG1021), LC539467(MS320), LC539542(MS353), LC539608(MS355), LC539692(MS358),

	LC539755(MS378), LC539869(MS444), LC539963(MS453), LC540054(ITS1), LC540144(ITS2), LC540238(mtLSU), LC540334(mtSSU)
F61	LC538448(FG546), LC538542(FG576), LC538618(FG652), LC538689(FG684), LC538779(FG756), LC538865(FG761), LC538956(FG771), LC539049(FG813), LC539142(FG848), LC539237(FG927), LC539333(FG975), LC539468(MS320), LC539543(MS353), LC539609(MS355), LC539693(MS358), LC539756(MS378), LC539796(MS417), LC539870(MS444), LC539964(MS453), LC540055(ITS1), LC540145(ITS2), LC540239(mtLSU), LC540335(mtSSU)
F62	LC538449(FG546), LC538543(FG576), LC538957(FG771), LC539050(FG813), LC539143(FG848), LC539238(FG927), LC539334(FG975), LC539469(MS320), LC539694(MS358), LC539871(MS444), LC539965(MS453), LC540056(ITS1), LC540146(ITS2), LC540240(mtLSU), LC540336(mtSSU)
F63	LC538450(FG546), LC538544(FG576), LC538619(FG652), LC538866(FG761), LC538958(FG771), LC539051(FG813), LC539144(FG848), LC539239(FG927), LC539335(FG975), LC539470(MS320), LC539610(MS355), LC539695(MS358), LC539872(MS444), LC539966(MS453), LC540057(ITS1), LC540147(ITS2), LC540241(mtLSU), LC540337(mtSSU)
F64	LC538451(FG546), LC538545(FG576), LC538620(FG652), LC538690(FG684), LC538780(FG756), LC538867(FG761), LC538959(FG771), LC539052(FG813), LC539145(FG848), LC539240(FG927), LC539336(FG975), LC539392(FG1021), LC539471(MS320), LC539544(MS353), LC539611(MS355), LC539696(MS358), LC539757(MS378), LC539873(MS444), LC539967(MS453), LC540058(ITS1), LC540148(ITS2), LC540242(mtLSU), LC540338(mtSSU)
F65	LC538452(FG546), LC538546(FG576), LC538621(FG652), LC538691(FG684), LC538781(FG756), LC538868(FG761), LC538960(FG771), LC539053(FG813), LC539146(FG848), LC539241(FG927), LC539337(FG975), LC539472(MS320), LC539545(MS353), LC539612(MS355), LC539697(MS358), LC539758(MS378), LC539874(MS444), LC539968(MS453), LC540059(ITS1), LC540149(ITS2), LC540243(mtLSU), LC540339(mtSSU)
F66	LC538453(FG546), LC538547(FG576), LC538622(FG652), LC538692(FG684), LC538782(FG756), LC538869(FG761), LC538961(FG771), LC539054(FG813), LC539147(FG848), LC539242(FG927), LC539338(FG975), LC539473(MS320), LC539613(MS355), LC539698(MS358), LC539759(MS378), LC539875(MS444), LC539969(MS453), LC540060(ITS1), LC540150(ITS2), LC540244(mtLSU), LC540340(mtSSU)
F67	LC538454(FG546), LC538548(FG576), LC538623(FG652), LC538693(FG684), LC538783(FG756), LC538870(FG761), LC538962(FG771), LC539055(FG813), LC539148(FG848), LC539243(FG927), LC539339(FG975), LC539474(MS320), LC539614(MS355), LC539699(MS358), LC539760(MS378), LC539876(MS444), LC539970(MS453), LC540245(mtLSU), LC540341(mtSSU)
F68	LC538455(FG546), LC538549(FG576), LC538624(FG652), LC538694(FG684), LC538784(FG756), LC538871(FG761), LC538963(FG771), LC539056(FG813), LC539149(FG848), LC539244(FG927), LC539340(FG975), LC539393(FG1021), LC539475(MS320), LC539546(MS353), LC539615(MS355), LC539700(MS358), LC539877(MS444), LC539971(MS453), LC540061(ITS1), LC540151(ITS2), LC540246(mtLSU), LC540342(mtSSU)
F69	LC538456(FG546), LC538550(FG576), LC538625(FG652), LC538695(FG684), LC538785(FG756), LC538872(FG761), LC538964(FG771), LC539057(FG813), LC539150(FG848), LC539245(FG927), LC539341(FG975), LC539476(MS320), LC539616(MS355), LC539701(MS358), LC539797(MS417), LC539878(MS444), LC539972(MS453), LC540062(ITS1), LC540152(ITS2), LC540247(mtLSU), LC540343(mtSSU)
F70	LC538457(FG546), LC538551(FG576), LC538626(FG652), LC538696(FG684), LC538786(FG756), LC538873(FG761), LC538965(FG771), LC539058(FG813), LC539151(FG848), LC539246(FG927), LC539342(FG975), LC539394(FG1021), LC539477(MS320), LC539617(MS355), LC539702(MS358), LC539761(MS378),

LC539798(MS417), LC539879(MS444), LC539973(MS453), LC540063(ITS1), LC540153(ITS2), LC540248(mtLSU), LC540344(mtSSU)

F71 LC539247(FG927), LC539343(FG975), LC539395(FG1021), LC539478(MS320), LC539703(MS358), LC539974(MS453), LC540064(ITS1), LC540154(ITS2), LC540249(mtLSU), LC540345(mtSSU)

F72 LC538458(FG546), LC538552(FG576), LC538627(FG652), LC538697(FG684), LC538787(FG756), LC538874(FG761), LC538966(FG771), LC539059(FG813), LC539152(FG848), LC539248(FG927), LC539344(FG975), LC539479(MS320), LC539618(MS355), LC539704(MS358), LC539880(MS444), LC539975(MS453), LC540065(ITS1), LC540155(ITS2), LC540250(mtLSU), LC540346(mtSSU)

F73 LC538459(FG546), LC538553(FG576), LC538628(FG652), LC538698(FG684), LC538788(FG756), LC538875(FG761), LC538967(FG771), LC539060(FG813), LC539153(FG848), LC539249(FG927), LC539345(FG975), LC539480(MS320), LC539619(MS355), LC539705(MS358), LC539762(MS378), LC539881(MS444), LC539976(MS453), LC540066(ITS1), LC540156(ITS2), LC540251(mtLSU), LC540347(mtSSU)

F74 LC538460(FG546), LC538554(FG576), LC538629(FG652), LC538699(FG684), LC538789(FG756), LC538876(FG761), LC538968(FG771), LC539061(FG813), LC539154(FG848), LC539250(FG927), LC539346(FG975), LC539481(MS320), LC539547(MS353), LC539620(MS355), LC539706(MS358), LC539763(MS378), LC539799(MS417), LC539882(MS444), LC539977(MS453), LC540067(ITS1), LC540157(ITS2), LC540252(mtLSU), LC540348(mtSSU)

F75 LC538461(FG546), LC538555(FG576), LC538630(FG652), LC538700(FG684), LC538790(FG756), LC538877(FG761), LC538969(FG771), LC539062(FG813), LC539155(FG848), LC539251(FG927), LC539347(FG975), LC539482(MS320), LC539548(MS353), LC539621(MS355), LC539707(MS358), LC539800(MS417), LC539883(MS444), LC539978(MS453), LC540068(ITS1), LC540253(mtLSU), LC540349(mtSSU)

F76 LC538556(FG576), LC538631(FG652), LC538701(FG684), LC538791(FG756), LC538970(FG771), LC539063(FG813), LC539156(FG848), LC539252(FG927), LC539348(FG975), LC539396(FG1021), LC539483(MS320), LC539549(MS353), LC539708(MS358), LC539764(MS378), LC539801(MS417), LC539884(MS444), LC539979(MS453), LC540069(ITS1), LC540158(ITS2), LC540254(mtLSU), LC540350(mtSSU)

F77 LC538462(FG546), LC538557(FG576), LC538632(FG652), LC538702(FG684), LC538792(FG756), LC538878(FG761), LC538971(FG771), LC539064(FG813), LC539157(FG848), LC539253(FG927), LC539349(FG975), LC539484(MS320), LC539550(MS353), LC539622(MS355), LC539709(MS358), LC539765(MS378), LC539885(MS444), LC539980(MS453), LC540070(ITS1), LC540159(ITS2), LC540255(mtLSU), LC540351(mtSSU)

F78 LC538463(FG546), LC538558(FG576), LC538703(FG684), LC538793(FG756), LC538972(FG771), LC539065(FG813), LC539158(FG848), LC539254(FG927), LC539350(FG975), LC539485(MS320), LC539623(MS355), LC539710(MS358), LC539766(MS378), LC539802(MS417), LC539886(MS444), LC539981(MS453), LC540071(ITS1), LC540160(ITS2), LC540256(mtLSU), LC540352(mtSSU)

F79 LC538464(FG546), LC538559(FG576), LC538633(FG652), LC538704(FG684), LC538794(FG756), LC538879(FG761), LC538973(FG771), LC539066(FG813), LC539159(FG848), LC539255(FG927), LC539351(FG975), LC539397(FG1021), LC539486(MS320), LC539551(MS353), LC539624(MS355), LC539711(MS358), LC539767(MS378), LC539803(MS417), LC539887(MS444), LC539982(MS453), LC540072(ITS1), LC540161(ITS2), LC540257(mtLSU), LC540353(mtSSU)

F80	LC538465(FG546), LC538560(FG576), LC538634(FG652), LC538705(FG684), LC538795(FG756), LC538974(FG771), LC539067(FG813), LC539160(FG848), LC539256(FG927), LC539352(FG975), LC539398(FG1021), LC539487(MS320), LC539712(MS358), LC539768(MS378), LC539804(MS417), LC539888(MS444), LC539983(MS453), LC540073(ITS1), LC540162(ITS2), LC540258(mtLSU), LC540354(mtSSU)
F81	LC538466(FG546), LC538561(FG576), LC538706(FG684), LC538796(FG756), LC538880(FG761), LC538975(FG771), LC539068(FG813), LC539161(FG848), LC539257(FG927), LC539353(FG975), LC539488(MS320), LC539625(MS355), LC539713(MS358), LC539889(MS444), LC539984(MS453), LC540074(ITS1), LC540163(ITS2), LC540259(mtLSU), LC540355(mtSSU)
F82	LC538467(FG546), LC538562(FG576), LC538707(FG684), LC538797(FG756), LC538881(FG761), LC538976(FG771), LC539069(FG813), LC539162(FG848), LC539258(FG927), LC539354(FG975), LC539399(FG1021), LC539489(MS320), LC539626(MS355), LC539714(MS358), LC539890(MS444), LC539985(MS453), LC540075(ITS1), LC540164(ITS2), LC540260(mtLSU), LC540356(mtSSU)
F83	LC538468(FG546), LC538563(FG576), LC538635(FG652), LC538708(FG684), LC538798(FG756), LC538882(FG761), LC538977(FG771), LC539070(FG813), LC539163(FG848), LC539259(FG927), LC539355(FG975), LC539400(FG1021), LC539490(MS320), LC539715(MS358), LC539891(MS444), LC539986(MS453), LC540076(ITS1), LC540165(ITS2), LC540261(mtLSU), LC540357(mtSSU)
F84	LC538469(FG546), LC538564(FG576), LC538636(FG652), LC538709(FG684), LC538799(FG756), LC538883(FG761), LC538978(FG771), LC539071(FG813), LC539164(FG848), LC539260(FG927), LC539356(FG975), LC539401(FG1021), LC539491(MS320), LC539892(MS444), LC539987(MS453), LC540077(ITS1), LC540166(ITS2), LC540262(mtLSU), LC540358(mtSSU)
F85	LC538470(FG546), LC538565(FG576), LC538710(FG684), LC538800(FG756), LC538884(FG761), LC538979(FG771), LC539072(FG813), LC539165(FG848), LC539261(FG927), LC539357(FG975), LC539402(FG1021), LC539492(MS320), LC539552(MS353), LC539627(MS355), LC539716(MS358), LC539769(MS378), LC539805(MS417), LC539893(MS444), LC539988(MS453), LC540078(ITS1), LC540167(ITS2), LC540263(mtLSU), LC540359(mtSSU)
F86	LC538471(FG546), LC538566(FG576), LC538637(FG652), LC538711(FG684), LC538801(FG756), LC538885(FG761), LC538980(FG771), LC539073(FG813), LC539166(FG848), LC539262(FG927), LC539358(FG975), LC539403(FG1021), LC539493(MS320), LC539717(MS358), LC539770(MS378), LC539894(MS444), LC539989(MS453), LC540079(ITS1), LC540168(ITS2), LC540264(mtLSU), LC540360(mtSSU)
F87	LC538472(FG546), LC538567(FG576), LC538638(FG652), LC538712(FG684), LC538802(FG756), LC538886(FG761), LC538981(FG771), LC539074(FG813), LC539167(FG848), LC539263(FG927), LC539359(FG975), LC539494(MS320), LC539628(MS355), LC539718(MS358), LC539771(MS378), LC539806(MS417), LC539895(MS444), LC539990(MS453), LC540080(ITS1), LC540169(ITS2), LC540265(mtLSU), LC540361(mtSSU)
F88	LC538473(FG546), LC538568(FG576), LC538639(FG652), LC538713(FG684), LC538803(FG756), LC538887(FG761), LC538982(FG771), LC539075(FG813), LC539168(FG848), LC539264(FG927), LC539360(FG975), LC539495(MS320), LC539719(MS358), LC539807(MS417), LC539896(MS444), LC539991(MS453), LC540081(ITS1), LC540170(ITS2), LC540266(mtLSU), LC540362(mtSSU)
F89	LC538474(FG546), LC538569(FG576), LC538640(FG652), LC538714(FG684), LC538804(FG756), LC538888(FG761), LC538983(FG771), LC539076(FG813), LC539169(FG848), LC539265(FG927), LC539361(FG975), LC539496(MS320), LC539553(MS353), LC539629(MS355), LC539720(MS358), LC539772(MS378), LC539808(MS417), LC539897(MS444), LC539992(MS453), LC540082(ITS1), LC540171(ITS2), LC540267(mtLSU), LC540363(mtSSU)

F90	LC538475(FG546), LC538570(FG576), LC538641(FG652), LC538715(FG684), LC538805(FG756), LC538889(FG761), LC538984(FG771), LC539077(FG813), LC539170(FG848), LC539266(FG927), LC539362(FG975), LC539497(MS320), LC539554(MS353), LC539630(MS355), LC539721(MS358), LC539809(MS417), LC539898(MS444), LC539993(MS453), LC540083(ITS1), LC540172(ITS2), LC540268(mtLSU), LC540364(mtSSU)
F91	LC538476(FG546), LC538571(FG576), LC538642(FG652), LC538716(FG684), LC538806(FG756), LC538890(FG761), LC538985(FG771), LC539078(FG813), LC539171(FG848), LC539267(FG927), LC539363(FG975), LC539404(FG1021), LC539498(MS320), LC539631(MS355), LC539722(MS358), LC539899(MS444), LC539994(MS453), LC540084(ITS1), LC540173(ITS2), LC540269(mtLSU), LC540365(mtSSU)
F92	LC538477(FG546), LC538572(FG576), LC538643(FG652), LC538717(FG684), LC538807(FG756), LC538891(FG761), LC538986(FG771), LC539079(FG813), LC539172(FG848), LC539268(FG927), LC539364(FG975), LC539405(FG1021), LC539499(MS320), LC539632(MS355), LC539723(MS358), LC539773(MS378), LC539900(MS444), LC539995(MS453), LC540085(ITS1), LC540174(ITS2), LC540270(mtLSU), LC540366(mtSSU)
F93	LC538478(FG546), LC538573(FG576), LC538644(FG652), LC538718(FG684), LC538808(FG756), LC538892(FG761), LC538987(FG771), LC539080(FG813), LC539173(FG848), LC539269(FG927), LC539365(FG975), LC539406(FG1021), LC539500(MS320), LC539724(MS358), LC539774(MS378), LC539810(MS417), LC539901(MS444), LC539996(MS453), LC540086(ITS1), LC540175(ITS2), LC540271(mtLSU), LC540367(mtSSU)
F94	LC538479(FG546), LC538574(FG576), LC538645(FG652), LC538719(FG684), LC538809(FG756), LC538893(FG761), LC538988(FG771), LC539081(FG813), LC539174(FG848), LC539270(FG927), LC539366(FG975), LC539407(FG1021), LC539501(MS320), LC539555(MS353), LC539633(MS355), LC539725(MS358), LC539775(MS378), LC539902(MS444), LC539997(MS453), LC540087(ITS1), LC540176(ITS2), LC540272(mtLSU), LC540368(mtSSU)
F95	LC538480(FG546), LC538575(FG576), LC538646(FG652), LC538720(FG684), LC538810(FG756), LC538894(FG761), LC538989(FG771), LC539082(FG813), LC539175(FG848), LC539271(FG927), LC539367(FG975), LC539502(MS320), LC539556(MS353), LC539634(MS355), LC539726(MS358), LC539776(MS378), LC539811(MS417), LC539903(MS444), LC539998(MS453), LC540088(ITS1), LC540177(ITS2), LC540273(mtLSU), LC540369(mtSSU)
yk334	LC538482(FG546), LC538577(FG576), LC538896(FG761), LC538991(FG771), LC539177(FG848), LC539273(FG927), LC539369(FG975), LC539408(FG1021), LC539504(MS320), LC540090(ITS1), LC540179(ITS2), LC540275(mtLSU), LC540371(mtSSU)
FS01	LC538481(FG546), LC538576(FG576), LC538811(FG756), LC538895(FG761), LC538990(FG771), LC539176(FG848), LC539272(FG927), LC539368(FG975), LC539503(MS320), LC539557(MS353), LC539727(MS358), LC539777(MS378), LC539812(MS417), LC539904(MS444), LC539999(MS453), LC540089(ITS1), LC540178(ITS2), LC540274(mtLSU), LC540370(mtSSU)

Table S3. Results of congruence among distance (CADM) matrices test for nuclear loci. For multiple comparison, P values were adjusted by the Hochberg correction.

Locus 1	Locus 2	W	Chi square	P	Adjusted P
FG1021	FG546	0.727	1076.2	0.0002	0.0231
FG1021	FG576	0.926	1442.9	0.0001	0.0231
FG1021	FG652	0.938	707.1	0.0001	0.0231
FG1021	FG684	0.897	1065.7	0.0001	0.0231
FG1021	FG756	0.935	1456.0	0.0001	0.0231
FG1021	FG761	0.899	1130.4	0.0001	0.0231
FG1021	FG771	0.924	1513.3	0.0001	0.0231
FG1021	FG813	0.929	1375.5	0.0001	0.0231
FG1021	FG848	0.935	1530.9	0.0001	0.0231
FG1021	FG927	0.935	1608.9	0.0001	0.0231
FG1021	FG975	0.932	1602.6	0.0001	0.0231
FG1021	ITS1	0.922	1585.3	0.0001	0.0231
FG1021	ITS2	0.931	1525.0	0.0001	0.0231
FG1021	MS320	0.934	1606.4	0.0001	0.0231
FG1021	MS353	0.918	346.9	0.0001	0.0231
FG1021	MS355	0.931	753.8	0.0001	0.0231
FG1021	MS358	0.880	1370.3	0.0001	0.0231
FG1021	MS378	0.837	384.9	0.0001	0.0231
FG1021	MS417	0.884	136.1	0.0033	0.0231
FG1021	MS444	0.930	1305.1	0.0001	0.0231
FG1021	MS453	0.886	1380.0	0.0001	0.0231
FG546	FG576	0.672	6391.1	0.0002	0.0231
FG546	FG652	0.598	2969.8	0.0073	0.0231
FG546	FG684	0.566	3309.2	0.0055	0.0231
FG546	FG756	0.661	5774.2	0.0001	0.0231
FG546	FG761	0.634	4849.7	0.0007	0.0231
FG546	FG771	0.673	6397.8	0.0001	0.0231
FG546	FG813	0.644	5631.4	0.0001	0.0231
FG546	FG848	0.648	6154.5	0.0002	0.0231
FG546	FG927	0.650	6175.7	0.0001	0.0231
FG546	FG975	0.649	6172.2	0.0001	0.0231
FG546	ITS1	0.673	5758.1	0.0001	0.0231
FG546	ITS2	0.675	5403.6	0.0001	0.0231
FG546	MS320	0.648	6154.5	0.0001	0.0231
FG546	MS353	0.638	1689.8	0.0029	0.0231
FG546	MS355	0.690	4357.0	0.0001	0.0231
FG546	MS358	0.601	5368.9	0.0018	0.0231
FG546	MS378	0.683	1672.6	0.0005	0.0231
FG546	MS417	0.801	952.0	0.0001	0.0231
FG546	MS444	0.645	5635.7	0.0001	0.0231
FG546	MS453	0.631	5877.6	0.0003	0.0231
FG576	FG652	0.980	5148.8	0.0001	0.0231
FG576	FG684	0.937	5624.2	0.0001	0.0231
FG576	FG756	0.965	8802.5	0.0001	0.0231
FG576	FG761	0.989	7573.2	0.0001	0.0231
FG576	FG771	1.000	9896.5	0.0001	0.0231
FG576	FG813	0.983	8961.3	0.0001	0.0231
FG576	FG848	0.964	9350.6	0.0001	0.0231
FG576	FG927	0.967	9568.3	0.0001	0.0231
FG576	FG975	0.976	9660.5	0.0001	0.0231

FG576	ITS1	0.996	8890.0	0.0001	0.0231
FG576	ITS2	0.989	8277.7	0.0001	0.0231
FG576	MS320	0.967	9567.0	0.0001	0.0231
FG576	MS353	0.983	2707.0	0.0001	0.0231
FG576	MS355	1.000	6317.5	0.0001	0.0231
FG576	MS358	0.901	8386.1	0.0001	0.0231
FG576	MS378	0.956	2436.3	0.0001	0.0231
FG576	MS417	0.960	1207.8	0.0001	0.0231
FG576	MS444	0.984	8968.9	0.0001	0.0231
FG576	MS453	0.973	9243.6	0.0001	0.0231
FG652	FG684	0.935	3888.0	0.0001	0.0231
FG652	FG756	0.975	4984.0	0.0001	0.0231
FG652	FG761	0.981	4336.0	0.0001	0.0231
FG652	FG771	0.989	5196.9	0.0001	0.0231
FG652	FG813	0.992	5211.3	0.0001	0.0231
FG652	FG848	0.968	4944.6	0.0001	0.0231
FG652	FG927	0.976	5130.0	0.0001	0.0231
FG652	FG975	0.995	5226.8	0.0001	0.0231
FG652	ITS1	0.986	4760.4	0.0001	0.0231
FG652	ITS2	0.989	4370.1	0.0001	0.0231
FG652	MS320	0.975	5124.7	0.0001	0.0231
FG652	MS353	0.977	1763.0	0.0001	0.0231
FG652	MS355	0.986	3605.8	0.0001	0.0231
FG652	MS358	0.948	4846.5	0.0001	0.0231
FG652	MS378	0.966	1661.4	0.0001	0.0231
FG652	MS417	0.956	774.7	0.0001	0.0231
FG652	MS444	0.987	5186.1	0.0001	0.0231
FG652	MS453	0.966	4936.1	0.0001	0.0231
FG684	FG756	0.966	5797.7	0.0001	0.0231
FG684	FG761	0.949	4581.0	0.0001	0.0231
FG684	FG771	0.942	5656.5	0.0001	0.0231
FG684	FG813	0.917	5366.2	0.0001	0.0231
FG684	FG848	0.966	5796.9	0.0001	0.0231
FG684	FG927	0.963	5784.3	0.0001	0.0231
FG684	FG975	0.923	5540.7	0.0001	0.0231
FG684	ITS1	0.942	5369.0	0.0001	0.0231
FG684	ITS2	0.938	4930.6	0.0001	0.0231
FG684	MS320	0.966	5799.5	0.0001	0.0231
FG684	MS353	0.979	1936.0	0.0001	0.0231
FG684	MS355	0.952	4083.3	0.0001	0.0231
FG684	MS358	0.984	5758.9	0.0001	0.0231
FG684	MS378	0.948	1792.3	0.0001	0.0231
FG684	MS417	0.954	1005.4	0.0001	0.0231
FG684	MS444	0.969	5669.8	0.0001	0.0231
FG684	MS453	0.916	5499.2	0.0001	0.0231
FG756	FG761	0.975	7127.3	0.0001	0.0231
FG756	FG771	0.989	9203.6	0.0001	0.0231
FG756	FG813	0.957	8546.4	0.0001	0.0231
FG756	FG848	0.991	9035.0	0.0001	0.0231
FG756	FG927	1.000	9306.6	0.0001	0.0231
FG756	FG975	0.978	9108.3	0.0001	0.0231
FG756	ITS1	0.952	7966.6	0.0001	0.0231
FG756	ITS2	0.963	7537.3	0.0001	0.0231

FG756	MS320	1.000	9308.3	0.0001	0.0231
FG756	MS353	0.991	2730.5	0.0001	0.0231
FG756	MS355	0.972	5834.6	0.0001	0.0231
FG756	MS358	0.956	8719.3	0.0001	0.0231
FG756	MS378	0.964	2455.2	0.0001	0.0231
FG756	MS417	0.960	1208.1	0.0001	0.0231
FG756	MS444	0.985	8611.2	0.0001	0.0231
FG756	MS453	0.948	8460.3	0.0001	0.0231
FG761	FG771	1.000	7652.3	0.0001	0.0231
FG761	FG813	0.981	7004.8	0.0001	0.0231
FG761	FG848	0.975	7464.4	0.0001	0.0231
FG761	FG927	0.975	7464.3	0.0001	0.0231
FG761	FG975	0.984	7529.6	0.0001	0.0231
FG761	ITS1	0.994	6763.0	0.0001	0.0231
FG761	ITS2	0.990	6254.1	0.0001	0.0231
FG761	MS320	0.975	7464.3	0.0001	0.0231
FG761	MS353	0.990	2137.7	0.0001	0.0231
FG761	MS355	1.000	5109.1	0.0001	0.0231
FG761	MS358	0.986	7202.8	0.0001	0.0231
FG761	MS378	0.960	1985.0	0.0001	0.0231
FG761	MS417	0.972	844.1	0.0001	0.0231
FG761	MS444	0.991	7238.6	0.0001	0.0231
FG761	MS453	0.977	7310.3	0.0001	0.0231
FG771	FG813	0.981	9130.9	0.0001	0.0231
FG771	FG848	0.988	9782.3	0.0001	0.0231
FG771	FG927	0.989	9987.2	0.0001	0.0231
FG771	FG975	0.984	9932.7	0.0001	0.0231
FG771	ITS1	0.988	9010.3	0.0001	0.0231
FG771	ITS2	0.991	8475.8	0.0001	0.0231
FG771	MS320	0.989	9988.6	0.0001	0.0231
FG771	MS353	0.992	2730.6	0.0001	0.0231
FG771	MS355	1.000	6317.5	0.0001	0.0231
FG771	MS358	0.930	8836.8	0.0001	0.0231
FG771	MS378	0.964	2455.1	0.0001	0.0231
FG771	MS417	0.969	1218.7	0.0001	0.0231
FG771	MS444	0.991	9039.1	0.0001	0.0231
FG771	MS453	0.972	9234.1	0.0001	0.0231
FG813	FG848	0.954	8703.0	0.0001	0.0231
FG813	FG927	0.960	8934.1	0.0001	0.0231
FG813	FG975	0.982	9142.9	0.0001	0.0231
FG813	ITS1	0.977	8176.4	0.0001	0.0231
FG813	ITS2	0.993	7772.5	0.0001	0.0231
FG813	MS320	0.958	8917.2	0.0001	0.0231
FG813	MS353	0.965	2556.2	0.0001	0.0231
FG813	MS355	0.981	5889.0	0.0001	0.0231
FG813	MS358	0.937	8546.6	0.0001	0.0231
FG813	MS378	0.958	2344.3	0.0001	0.0231
FG813	MS417	0.961	1141.7	0.0001	0.0231
FG813	MS444	0.984	8599.3	0.0001	0.0231
FG813	MS453	0.967	8634.3	0.0001	0.0231
FG848	FG927	1.000	9894.9	0.0001	0.0231
FG848	FG975	0.977	9674.6	0.0001	0.0231
FG848	ITS1	0.950	8482.8	0.0001	0.0231

FG848	ITS2	0.969	8108.7	0.0001	0.0231
FG848	MS320	1.000	9897.9	0.0001	0.0231
FG848	MS353	0.992	2730.7	0.0001	0.0231
FG848	MS355	0.968	6113.4	0.0001	0.0231
FG848	MS358	0.960	8933.6	0.0001	0.0231
FG848	MS378	0.964	2455.0	0.0001	0.0231
FG848	MS417	0.969	1218.8	0.0001	0.0231
FG848	MS444	0.984	8781.8	0.0001	0.0231
FG848	MS453	0.952	9046.6	0.0001	0.0231
FG927	FG975	0.981	10103.2	0.0001	0.0231
FG927	ITS1	0.955	8886.6	0.0001	0.0231
FG927	ITS2	0.975	8522.6	0.0001	0.0231
FG927	MS320	1.000	10297.0	0.0001	0.0231
FG927	MS353	0.992	2730.7	0.0001	0.0231
FG927	MS355	0.995	6286.4	0.0001	0.0231
FG927	MS358	0.955	9265.8	0.0001	0.0231
FG927	MS378	0.964	2455.2	0.0001	0.0231
FG927	MS417	0.969	1218.7	0.0001	0.0231
FG927	MS444	0.985	8977.8	0.0001	0.0231
FG927	MS453	0.954	9252.7	0.0001	0.0231
FG975	ITS1	0.963	8961.9	0.0001	0.0231
FG975	ITS2	0.992	8670.6	0.0001	0.0231
FG975	MS320	0.979	10087.3	0.0001	0.0231
FG975	MS353	0.991	2730.5	0.0001	0.0231
FG975	MS355	0.968	6116.3	0.0001	0.0231
FG975	MS358	0.901	8743.4	0.0001	0.0231
FG975	MS378	0.964	2455.1	0.0001	0.0231
FG975	MS417	0.960	1208.1	0.0001	0.0231
FG975	MS444	0.984	8974.0	0.0001	0.0231
FG975	MS453	0.968	9390.2	0.0001	0.0231
ITS1	ITS2	0.990	8649.1	0.0001	0.0231
ITS1	MS320	0.955	8887.7	0.0001	0.0231
ITS1	MS353	0.991	2329.5	0.0001	0.0231
ITS1	MS355	1.000	5546.5	0.0001	0.0231
ITS1	MS358	0.881	7701.5	0.0001	0.0231
ITS1	MS378	0.969	2183.7	0.0001	0.0231
ITS1	MS417	0.968	1150.2	0.0001	0.0231
ITS1	MS444	0.990	8109.7	0.0001	0.0231
ITS1	MS453	0.967	8453.6	0.0001	0.0231
ITS2	MS320	0.974	8513.7	0.0001	0.0231
ITS2	MS353	0.991	2233.8	0.0001	0.0231
ITS2	MS355	0.994	5080.5	0.0001	0.0231
ITS2	MS358	0.898	7349.8	0.0001	0.0231
ITS2	MS378	0.969	2183.7	0.0001	0.0231
ITS2	MS417	0.968	1084.1	0.0001	0.0231
ITS2	MS444	0.996	7620.7	0.0001	0.0231
ITS2	MS453	0.976	7990.7	0.0001	0.0231
MS320	MS353	0.991	2730.5	0.0001	0.0231
MS320	MS355	0.968	6113.3	0.0001	0.0231
MS320	MS358	0.958	9291.0	0.0001	0.0231
MS320	MS378	0.964	2455.0	0.0001	0.0231
MS320	MS417	0.968	1218.0	0.0001	0.0231
MS320	MS444	0.985	8980.1	0.0001	0.0231

MS320	MS453	0.952	9238.4	0.0001	0.0231
MS353	MS355	0.990	2521.8	0.0001	0.0231
MS353	MS358	0.992	2730.7	0.0001	0.0231
MS353	MS378	0.990	1316.1	0.0001	0.0231
MS353	MS417	0.968	487.7	0.0001	0.0231
MS353	MS444	0.991	2730.5	0.0001	0.0231
MS353	MS453	0.991	2730.5	0.0001	0.0231
MS355	MS358	0.975	6005.1	0.0001	0.0231
MS355	MS378	0.991	1960.2	0.0001	0.0231
MS355	MS417	0.964	780.5	0.0001	0.0231
MS355	MS444	1.000	5849.8	0.0001	0.0231
MS355	MS453	0.966	6102.4	0.0001	0.0231
MS358	MS378	0.963	2454.9	0.0001	0.0231
MS358	MS417	0.968	1218.1	0.0001	0.0231
MS358	MS444	0.945	8434.6	0.0001	0.0231
MS358	MS453	0.941	8765.1	0.0001	0.0231
MS378	MS417	0.932	604.1	0.0001	0.0231
MS378	MS444	0.964	2455.0	0.0001	0.0231
MS378	MS453	0.930	2370.5	0.0001	0.0231
MS417	MS444	0.968	1218.0	0.0001	0.0231
MS417	MS453	0.936	1177.8	0.0001	0.0231
MS444	MS453	0.961	8579.2	0.0001	0.0231
